# Research on expressway traffic accident risk prediction method considering Spatio-Temporal effects

**DOI:** 10.1371/journal.pone.0360312

**Published:** 2026-10-23

**Authors:** Liang Zhao, Duo Liu, Han Bai, Tianzheng Wei, Jian Lu, Xiuguang Wang

**Affiliations:** 1 School of Transportation and Logistics Engineering, Shandong Jiaotong University, Jinan, China; 2 Shandong Zhengqu Traffic Engineering Co., Ltd, Jinan, China; Beijing Institute of Technology, CHINA

## Abstract

Traffic accidents arise from the dynamic evolution of traffic flow under spatio-temporal effects, and their occurrence and severity are significantly influenced by temporal and spatial variations in traffic conditions. Traditional studies predominantly rely on static accident-time data, which limits prediction accuracy. To address this gap, this study proposes a deep learning framework that integrates data preprocessing, spatio-temporal effect analysis, PCA-FCM-based risk labeling, and a CNN-BiLSTM hybrid model. Compared with CNN-LSTM, the proposed model replaces unidirectional LSTM with bidirectional LSTM to capture both forward and backward temporal dependencies. Based on the HighD naturalistic trajectory dataset, the CNN-BiLSTM model achieves an MSE of 0.362, RMSE of 0.601, MAE of 0.197 and MAPE of 0.044, outperforming LSTM, BiLSTM and CNN-LSTM with moderate running time. In accident risk classification, it reaches an overall accuracy of 90%, with class-level precision of 0.90–0.93 and F1 scores of 0.82–0.96. The results demonstrate that the proposed method effectively captures spatio-temporal dynamic characteristics and provides an efficient tool for expressway accident risk prediction.

## Introduction

With the vigorous development of the automobile industry, the demand for transportation travel is increasingly strong. Expressways are characterized by large traffic flow and complex vehicle composition, resulting in relatively high risk and loss of traffic safety accidents. Expressway traffic accidents are a common problem around the world, posing a serious threat to people’s lives and property. Therefore, predicting the occurrence of different types of traffic accidents on expressways is of great significance for taking corresponding prevention and rescue measures.

Domestic and foreign experts have conducted in-depth research on traffic accident risk. By deeply analyzing the internal laws of traffic accidents, researchers can construct a scientific and reasonable analysis framework to address the prominent problems urgently to be solved in the current road safety field. Piccinini et al. [1] analyzed the influencing factors of commercial vehicles in rear-end accidents through on-board event data recording. Seppelt et al. [2] studied the accident risk of critical accidents using videos from the Virginia Tech Transportation Institute. Gu et al. [3] studied the accident risk at interchange merging areas using UAV video data. Traditional studies mainly rely on questionnaire surveys or laboratory simulated driving experiments, but questionnaire survey data have biases, and laboratory simulation is difficult to restore real traffic environments. In contrast, natural driving datasets come from real traffic scenarios, can comprehensively reflect the impact of driving behavior, traffic flow characteristics and environmental factors on accident risk, and provide a more reliable scientific basis for revealing accident distribution laws, development trends and accident mechanisms.

Research on influencing factors of traffic accident risk covers multiple dimensions such as traffic flow characteristics, road characteristics, environmental characteristics and driving behavior. In terms of traffic flow stability and safety, Du Wenju et al. [4] studied complex mixed traffic flow composed of manually driven vehicles, autonomous vehicles, connected automated vehicles and connected manually driven vehicles, and found that considering information from multiple front and rear vehicles significantly improves traffic flow stability and safety, with the best effect when considering information from two front and rear vehicles. Yao et al. [5] further pointed out that the stability of mixed traffic flow increases with the increase of steady-state speed, but the stability is poor when the penetration rate of CAV (Connected Automated Vehicles) is low. In terms of speed dispersion and accident risk, Fu Qiang [6] introduced speed dispersion description parameters and found that traffic flow stability is significantly affected by speed dispersion rate. When the required safety distance is greater than the actual vehicle distance, the higher the car-following speed, the more likely the vehicle is in an unsafe state. Zhang Ruiqin et al. [7] pointed out that the average road speed and the number of different vehicle types affect the degree of speed dispersion, thereby affecting the probability of accidents. In terms of traffic flow modeling and prediction, Naheliya et al. [8] developed a complex framework for short-term traffic flow prediction and verified its accuracy through error evaluation methods. Yang Yang et al. [9] improved the performance of real-time accident risk prediction models on expressways by constructing multi-scale datasets and adopting Bayesian updating. Lin Heng et al. [10] established two interpretable traffic flow stability coefficients, simplified the index system, improved practicability and enhanced the reliability of accident risk prediction models.

Road geometric characteristics and alignment design are core factors affecting traffic accident risk. Based on describing road alignment with feature points, Pei Boli et al. [11] explored a representation method that best approximates alignment feature points, constructed a data structure model of road alignment, and studied the impact of road conditions on traffic accidents. Qi et al. [12] proposed the Traffic Order Index (TOI) to assess safety risks and found that congestion index, number of lanes, guide signs and weather conditions have a significant impact on traffic order. Road network characteristics and accident causation mechanism are key areas of traffic accident research. Xiao Yangmou et al. [13] found that the positive impact of increasing road network density outweighs the negative impact, which can effectively improve road traffic safety. Li Jiankan et al. [14] found through statistical analysis that urban road traffic accidents mostly occur at road sections, and the number of accidents is concentrated on Fridays and during morning and evening peaks. Gao Yinan et al. [15] analyzed 109 accident data and revealed essential factors such as speeding, overloading, technical defects or lagging regulations and standards, providing key points for improving accident risk.

Among environmental characteristics, weather conditions and visibility have a significant impact on accident risk. Severe weather weakens driver visibility and vehicle grip, increasing accident probability. Lin Zhongling et al. [16] showed that rainfall is a key risk factor affecting road traffic safety, which adversely affects human, vehicle, road and environmental elements in the traffic system, thereby increasing driving risk and accident rate. Wen et al. [17] found that monthly average wind speed, monthly average daily precipitation and monthly average visibility are significantly positively correlated with accident frequency. Most existing studies focus on static data at the moment of accidents, treating accidents as a single segment, which cannot fully reflect the dynamic evolution of traffic flow and traffic conditions before and after accidents. Therefore, this study proposes to re-examine traffic accidents from the perspective of spatio-temporal effects, emphasizing that accident prediction should comprehensively consider dynamic traffic conditions before and after accidents.

In the research of traffic accident risk prediction, existing studies still face challenges related to data quality, model complexity, and the diversity of application scenarios. For traditional statistical methods, Zhang Cuiping [18] comprehensively employed descriptive statistical analysis, regression analysis, discriminant analysis, model comparison and validation, and spatial statistical analysis to reveal the underlying patterns of accident occurrence. Although traditional methods, such as logistic regression and support vector machines, are relatively simple and easy to implement, they have obvious limitations in capturing dynamic spatio-temporal characteristics. With the rapid development of artificial intelligence and spatio-temporal learning, recent traffic prediction studies have increasingly focused on dynamic graph, attention-based, and hybrid deep-learning architectures. Ali et al. [19] developed a dynamic spatio-temporal framework and a GCN-enhanced variant to jointly model spatial dependencies, multiple temporal granularities, and external factors for citywide crowd-flow prediction. Ali et al. [20]further incorporated multi-scale dynamic graph convolution, temporal attention, and weather-aware cross-modal fusion, demonstrating the value of adaptive multimodal representations for modeling complex traffic dynamics. Ali et al. [21] addressed sparse neighborhood connectivity and long-term inter-node dependencies through dynamic multi-graph learning, while Ali et al. [22] employed attention mechanisms to model dynamic spatio-temporal correlations and different temporal patterns. Ali et al. [23] further explored data aggregation and fog-computing deployment, showing that removing redundant information from related time series can improve both prediction speed and accuracy. These studies demonstrate the advantages of graph-based, attention-based, and data-aggregation strategies for learning evolving spatio-temporal relationships. However, their primary focus is citywide traffic- or crowd-flow forecasting rather than vehicle-level accident-risk classification. Moreover, such models generally require more elaborate graph construction, multi-branch fusion, or attention mechanisms, and their effectiveness in capturing the microscopic evolution of vehicle interactions preceding expressway crashes remains insufficiently established. Ali et al. [24] reviewed the development of traffic prediction methods from convolutional and recurrent models to graph-based and attention-driven spatio-temporal networks, and highlighted continuing challenges related to generalization, heterogeneous data distributions, explainability, and resource-aware deployment. Therefore, simply increasing model complexity does not necessarily address the specific challenges of vehicle-level accident-risk prediction.

Methodological evidence from other application domains further highlights several issues relevant to the present study. Alsarhan et al. [25] proposed a lightweight Gated-Inception model that adaptively regulates information flow across different branches and suppresses redundant activations, suggesting that selective feature fusion can improve discriminative robustness while controlling parameter growth. Yang et al. [26] combined noise suppression, variational mode decomposition, and autoencoder-based feature extraction to address low-sampling-rate and noisy sensing conditions, emphasizing the importance of robust representation learning for real-world data. Sharafian et al. [27] introduced an adaptive RBF neural network within a sliding-mode control framework to estimate uncertainties and compensate for dynamic disturbances, illustrating the importance of explicitly considering uncertainty in complex dynamic systems. Ali et al. [28] addressed computational-resource constraints through energy-efficient virtual-machine allocation, showing that algorithmic performance should also be considered together with computational efficiency in large-scale deployment. Although these cross-domain studies do not directly address traffic accident-risk prediction, their findings reinforce the importance of robust feature extraction, selective fusion of informative variables, consideration of uncertainty, and a reasonable balance between predictive accuracy and computational cost. Taken together with the recent traffic-prediction literature, three gaps remain particularly important for expressway accident-risk prediction. First, most advanced spatio-temporal graph and attention-based models focus on regional traffic-flow forecasting rather than the dynamic evolution of individual vehicle interactions. Second, network-level spatial representations do not directly encode microscopic safety variables such as following distance, relative speed, acceleration difference, lane-changing behavior, and time to collision. Third, multimodal and highly adaptive architectures can increase data and computational requirements, whereas naturalistic trajectory datasets may not contain information on weather, infrastructure, or driver-specific characteristics. These gaps motivate the present study to use the HighD naturalistic driving dataset to characterize accident risk from vehicle-level spatio-temporal interactions and to investigate whether a relatively compact CNN-BiLSTM architecture can capture local feature patterns and temporal dependencies while maintaining practical computational efficiency. On this basis, PCA-FCM is used to reduce feature collinearity and construct risk categories from multiple traffic-interaction and surrogate-safety indicators, and the resulting labels are used for supervised prediction. The CNN-BiLSTM framework is then optimized using time-series cross-validation and grid search, so that the model design is guided by a balance among spatio-temporal representation capability, heterogeneous feature robustness, and model complexity, rather than simply adopting a more computationally demanding graph- or attention-based architecture.

Traffic accident risk evolution is a complex process involving continuous changes in vehicle interaction states and temporal dependencies before accidents. Therefore, prediction methods should not only identify temporal patterns but also capture the spatial interaction characteristics among vehicles. However, traditional risk assessment methods based on single surrogate safety indicators or manually defined thresholds may not fully represent the comprehensive risk states of traffic interactions. To overcome these limitations, this study introduces PCA-FCM to generate data-driven risk categories by integrating multiple traffic-interaction features and reducing feature redundancy. Furthermore, considering that accident risk contains both spatial interaction patterns and temporal evolution characteristics, a CNN-BiLSTM framework is proposed to extract spatial features through CNN and capture long-term temporal dependencies through BiLSTM. The proposed framework provides a systematic approach to expressway accident-risk prediction by integrating risk classification with spatio-temporal feature learning. This study mainly focuses on the following three aspects:

(1) Combining time and space dimensions, analyze the spatio-temporal interaction of traffic accident risk and extract high-incidence related features of accidents under specific time and space conditions.
(2) Divide accident risk into different levels: minor accidents, general accidents and serious accidents through cluster analysis, providing clear target variables for model construction.
(3) Propose a CNN-BiLSTM hybrid model, use CNN to extract spatial features of traffic data, capture long-term dependencies in time series through BiLSTM, and determine the optimal parameter configuration of the model using cross-validation and grid search methods.

## Materials and methods

This study conducts a full-process research on expressway traffic accident risk prediction, and the overall method system covers four core links: data extraction and preprocessing, spatio-temporal effect analysis and risk classification, prediction model construction and optimization, model application and evaluation, forming a closed-loop research from data processing to model verification step by step.

### Data extraction and preprocessing

This section focuses on the acquisition and processing of natural driving data, systematically introducing the basis for dataset selection, risk determination methods and preprocessing processes. Firstly, by comparing the characteristics and limitations of mainstream natural driving datasets, the applicability of the German HighD dataset in this study is clarified. Secondly, a traffic accident risk determination framework is constructed based on Surrogate Safety Measures (SSM), and a multi-dimensional data extraction method integrating Time to Collision (TTC) and Time Headway (THW) is proposed. Finally, aiming at the noise, redundancy and structural problems in the original data, a data cleaning and integration strategy is designed to ensure the accuracy and reliability of subsequent modeling analysis. Based on position information, it is divided into 60 parts, numbered from 1 to 60. Each data set includes high-altitude images, metadata, bicycle tracking information, and trajectory information per frame. As shown in Table 1.

**Table 1:** Main data of the HighD dataset.

File Name	Data Information
xx_ highway	Image information of the shooting section
xx_ recording Meta	Location information
	Shooting time
	Continuous shooting distance and time
	Number of vehicles (cars and trucks)
	Real-time vehicle speed
	Real-time vehicle acceleration
	Relative position relationship between vehicles
	Safety evaluation indicators between vehicles
xx_ tracks Meta	Vehicle size
	Start and end time of the trip
	Driving direction
	Statistical values of driving characteristics
	Vehicle lane change information

Surrogate Safety Measures (SSM) is a widely used performance indicator in Traffic Conflict Techniques (TCT). It indirectly evaluates traffic safety performance by analyzing traffic conflict events. A variety of safety evaluation performance indicators can be obtained based on SSM. Considering vehicle data conditions and the integrity of evaluation data, TTC and THW are selected, as shown in Formula 1 and Formula 2 below.


$$\begin{array}{c} \begin{array}{c} THW=\frac{DHW}{v_{\text{rear}}} \end{array} \end{array}$$ (1)

$$\begin{array}{c} \begin{array}{c} TTC=\frac{DHW}{v_{\text{rear}}-v_{\text{front}}} \end{array} \end{array}$$ (2)

Referring to the scenario threat assessment method based on the dataset by Patrick Schneider [29] and combined with the classification standard of expressway traffic conflicts by Jie Wenqi [30], this paper adopts TTC < 4s and THW < 2s as the extraction conditions for high-risk events. TTC < 4s is used to screen potential conflict events, and THW < 2s is used to ensure that the following distance is within a dangerous range. This combined threshold can effectively balance recall rate and precision rate.

Importantly, TTC < 4 s and THW < 2 s are used only to identify candidate high-risk interaction events and are not used as direct criteria for assigning the final risk categories. The subsequent risk labels are derived from the joint characteristics of multiple traffic-interaction variables through the PCA-FCM procedure described in Section 2.2.2

Due to the large number of entries in some files, software such as Excel may omit data, so Python is used for processing and screening. The extraction process is shown in Table 2.

**Table 2:** Accident risk data extraction process.

Step	Description
1.	Import pandas and os libraries
2.	TTC_THRESHOLD = 4 THW_THRESHOLD = 2
3.	Set data_directory path -Get list of all files all_files -Iterate through files and read as DataFrame
4.	Iterate through each DataFrame -Filter rows where TTC < 4 and THW < 2 - Store results in accident_data
5.	Merge accident_data - Save as accident_data.csv - If no data, output a message

Aiming at the noise and structural problems in the original data, multi-dimensional data cleaning is carried out: eliminate invalid data such as negative THW values, congested vehicles with speed lower than 40 km/h, and trips with duration less than 360 seconds, convert the speed of two-way traffic flow to absolute value and correct the acceleration sign.

After the data cleaning process, this study retains valid data samples, and each sample completely records multi-dimensional characteristic parameters related to traffic safety events. However, since the data collection system adopts a distributed recording architecture, each file independently performs spatio-temporal sampling and feature calculation. In the data fusion stage, cross-file conflicts of time frame identifiers (frame) and vehicle unique identification codes (id) are found, specifically manifested as repeated sequence numbers and timing misalignment. To eliminate identification ambiguity in data integration, this study constructs a globally consistent data index system. By inserting a file serial number column, reordering id and frame, and implementing cross-sample spatio-temporal remapping and entity coding standardization, each traffic entity has a unique traceable identification code in the global spatio-temporal framework, realizing data coherence and consistency.

### Spatio-temporal effect analysis and classification of traffic accident risk

Expressway traffic accident risk has significant spatio-temporal heterogeneity characteristics, and its formation mechanism and evolution law are affected by the dual effects of temporal dynamics and spatial differences. Based on high-precision natural driving data, this chapter systematically explores the spatio-temporal distribution characteristics and internal correlation mechanism of traffic accident risk using multi-dimensional analysis methods. Firstly, a spatio-temporal effect analysis framework is constructed theoretically to clarify the interaction mechanism of temporal and spatial dimensions on accident risk. Secondly, empirical analysis reveals the distribution law and evolution characteristics of accident risk under specific spatio-temporal conditions. Finally, a spatio-temporal feature-integrated accident risk classification model is constructed to achieve accurate identification of different risk levels. The research results will provide a theoretical basis and technical support for active safety prevention and control of expressways, and are of great significance for improving road traffic safety management.

#### Spatio-temporal effect analysis of traffic accident risk.

Expressway traffic accident risk has significant spatio-temporal effect characteristics. Based on the HighD dataset, this section conducts empirical research from two dimensions: temporal effect and spatial effect. 17 characteristic indicators such as rear vehicle speed/acceleration, headway, time to collision and number of lane changes are extracted, and precise extraction of key parameters such as rear vehicle speed/acceleration and front vehicle acceleration is realized through Python. Descriptive statistics, kernel density distribution analysis and Pearson correlation coefficient analysis are used to mine features, and significant collinearity problems are found between variables. Finally, 10 core features without obvious collinearity are selected.

(1) Feature Selection

To study traffic accident risk, surrogate safety indicators are considered. According to Formula (3), headway is directly related to the speed of front and rear vehicles, so this indicator is extracted. In addition, through literature review and expert experience, common characteristic indicators of traffic accident risk are obtained, including the average speed of the rear vehicle, average acceleration of the rear vehicle, average headway, standard deviation of headway, totaling 17 items, which are classified and sorted in Table 3.

**Table 3:** Feature extraction table.

Data Label	Description	Definition label
Vx_mean	Mean speed of rear vehicle	*X*_1_
Vx_std	Standard deviation of mean speed of rear vehicle	*X*_2_
Ax_mean	Mean acceleration of rear vehicle	*X*_3_
Ax_std	Standard deviation of acceleration of rear vehicle	*X*_4_
dhw_mean	Mean headway	*X*_5_
dhw_std	Standard deviation of headway	*X*_6_
ttc_mean	Mean time to collision	*X*_7_
ttc_std	Standard deviation of time to collision	*X*_8_
pVx_mean	Mean speed of front vehicle	*X*_9_
pVx_std	Standard deviation of speed of front vehicle	*X*_10_
pAx_mean	Mean acceleration of front vehicle	*X*_11_
pAx_std	Standard deviation of acceleration of front vehicle	*X*_12_
nLC_mean	Number of lane changes	*X*_13_
sV_mean	Mean speed difference between front and rear vehicles	*X*_14_
sV_std	Standard deviation of speed difference between front and rear vehicles	*X*_15_
sA_mean	Mean acceleration difference between front and rear vehicles	*X*_16_
sA_std	Standard deviation of acceleration difference between front and rear vehicles	*X*_17_

The calculation of relevant indicators is as follows:


$$\begin{array}{c} \begin{array}{c} {dhw}_{j,mean}=\frac{\left[\sum_{i=\text{1}}^{n}{(S}_{ij\text{front}}-S_{ij\text{rear}})\right]}{n} \end{array} \end{array}$$ (3)

${dhw}_{j,mean}$ represents the average headway of the j-th vehicle; $S_{ijfront}$ represents the lateral distance of the front vehicle of the j-th vehicle at the i-th frame,$S_{ijrear}$ represents the lateral distance of the j-th vehicle at the i-th frame; n represents the number of frames.

In the process of indicator extraction, considering the accuracy and efficiency of indicator extraction, Python is used for extraction, and a method to extract the acceleration of the front vehicle corresponding to dangerous following vehicles is proposed. Firstly, time series alignment of vehicles in dual datasets is realized through merge operation to establish a spatio-temporal correlation benchmark. Secondly, a dynamic following topology network is constructed based on the mapping relationship between id and preceding Id in dangerous following data. Finally, under the constraint of time frame alignment, dual trajectories are synchronized to extract the xAcceleration parameter of the target front vehicle at the moment of dangerous following. Details are shown in Table 4.

**Table 4:** Extracting the acceleration pseudocode of the vehicle in front.

Step	Description
**1.**	Load two CSV files into Data Frames named“df1”and“df2”.
**2.**	Merge df1 and df2 on ‘precedingId’ (in df1) and ‘id’ (in df2) using a left join. < br>Retain columns ‘frame,’ ‘id,’ and ‘xAcceleration’ from df2.
**3.**	Verify if the ‘frame’ column is renamed to ‘frame_x’ and ‘frame_y’ after merging. If so, ensure they match.
**4.**	Filter rows where the ‘frame’ column matches, generating result_df_filtered.
**5.**	Print the contents of result_df_filtered.
**6.**	Save result_df_filtered to a new CSV file.

A multi-indicator calculation method with higher efficiency than normal calculation is proposed. The calculation process of each indicator is shown in Table 5.

**Table 5:** Calculate the mean and standard deviation pseudocode of each indicator.

Step	Description
**1.**	mport the pandas library.
**2.**	Load the CSV file from a specified path into a DataFrame using pan-das.read_csv.
**3.**	Group the DataFrame by the ‘id’ column.
**4.**	For each group, calculate the mean and standard deviation of the following columns: <br>- “xVelocity<br > - xAcceleration<br > - dhw < br > - ttc < br > - precedingXVelocity<br > -speeddifference<br > - xAcceleration_df2
**5.**	(Optional) Reset the index of the grouped DataFrame for easier viewing and processing.
**6.**	Print the calculated statistical results.
	Save the statistical results to a new CSV file.

(2) Feature Analysis
  1) Descriptive Statistics

The quartile table is a descriptive statistical tool based on quartiles, used to describe the distribution characteristics of data, especially the central position and dispersion degree of data. Quartiles divide data into four equal parts, each containing the same number of data points, as shown in Tables 6 and 7.

**Table 6:** Quartile table.

	X1	X2	X3	X4	X5	X6	X7	X8	X9
**mean**	32.18	0.14	0.18	0.05	18.92	1.59	3.38	0.31	26.61
**std**	4.82	0.27	0.68	0.10	10.93	1.69	0.45	0.21	4.01
**min**	10.17	0.00	−5.52	0.00	2.86	0.02	1.06	0.00	7.46
**25%**	29.06	0.03	−0.06	0.01	10.48	0.45	3.14	0.14	24.02
**50%**	32.29	0.07	0.24	0.02	16.17	0.98	3.47	0.28	25.69
**75%**	35.18	0.15	0.55	0.05	24.90	2.15	3.74	0.44	29.15
**max**	52.45	4.29	2.78	1.28	69.69	14.33	3.99	1.05	41.89

**Table 7:** Quartile table.

	X10	X11	X12	X13	X14	X15	X16	X17
**mean**	0.08	−0.07	0.03	0.97	5.57	0.14	0.26	0.06
**std**	0.21	0.40	0.05	0.53	3.11	0.23	0.59	0.12
**min**	0.00	−4.52	0.00	0.00	1.01	0.00	−3.44	0.00
**25%**	0.01	−0.18	0.01	1.00	3.23	0.03	−0.01	0.01
**50%**	0.03	−0.04	0.01	1.00	4.74	0.07	0.29	0.02
**75%**	0.07	0.12	0.02	1.00	7.23	0.17	0.59	0.06
**max**	3.16	1.12	0.61	3.00	22.79	2.72	3.87	1.79

According to Fig 1, some values of variables X2, X4, X6, X10, X12, X15 and X17 are significantly lower than others, and the value of X7 is significantly higher than others, indicating that there may be outliers or particularities in the data, which require outlier processing or data normalization. Other variables generally show a certain concentration, and most of them have no significant outliers or extreme values. The stability of these variables can provide reliable data support to help formulate effective strategies.

**Fig 1:**
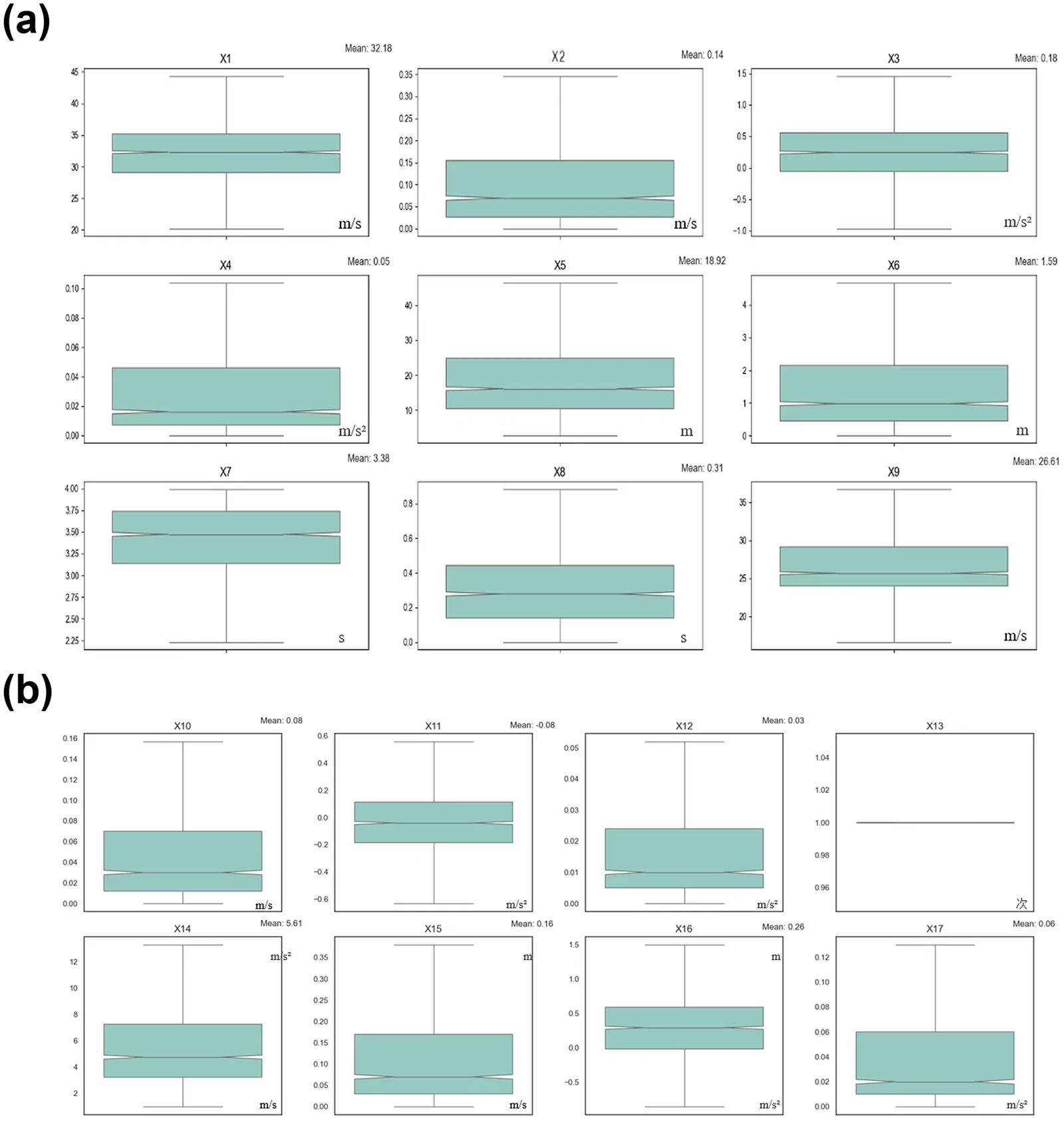
X1-X17 Quadrant chart.

2) Distribution characteristics

Studying the data distribution is a key step in data analysis, helping to understand the internal laws and characteristics of data. To show the data distribution more intuitively, kernel density distribution is adopted, as shown in Fig 2.

**Fig 2:**
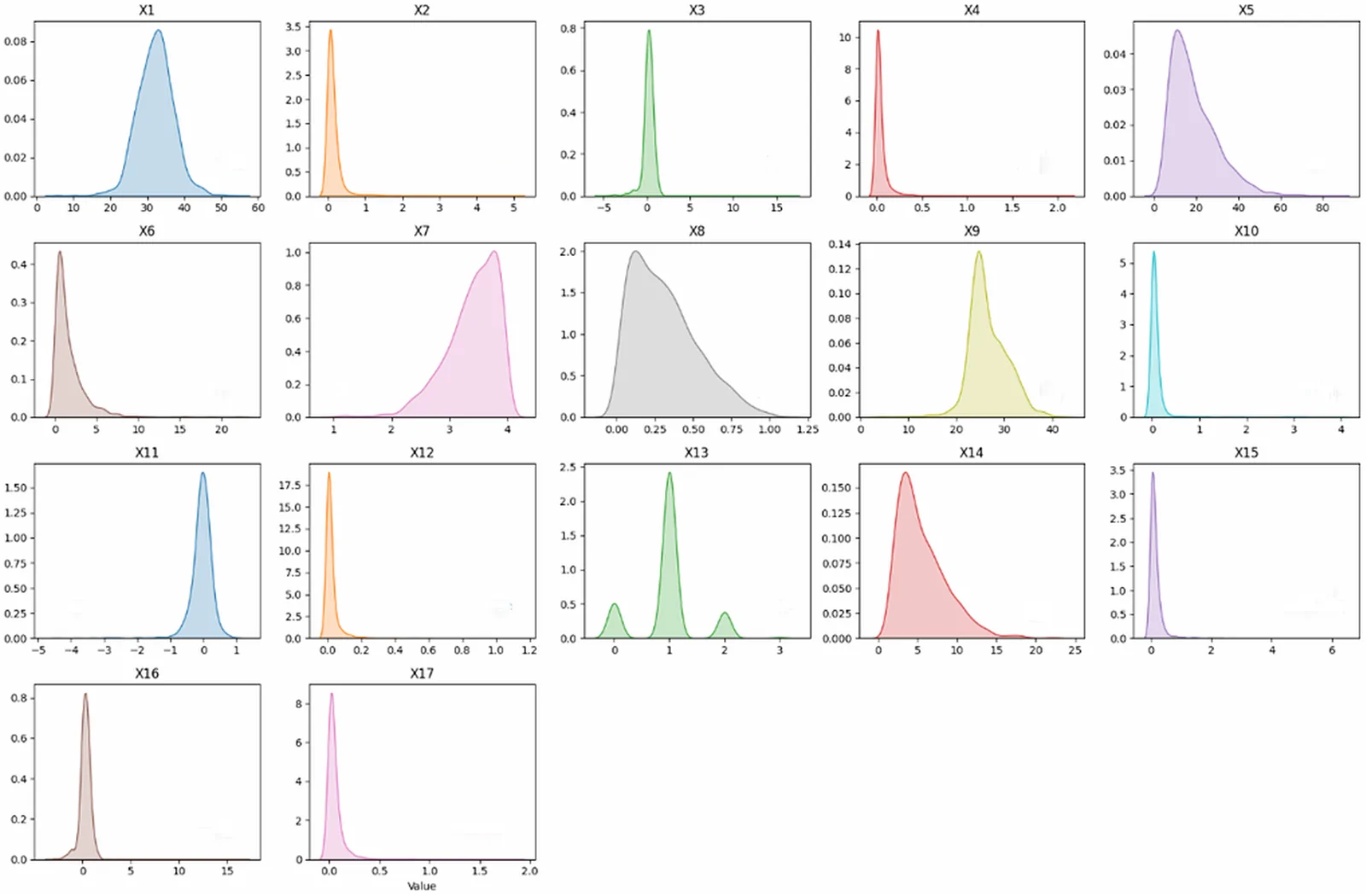
X1-X17 kernel density distribution.

According to Fig 2, X1 presents a unimodal normal distribution shape with low dispersion; X2 presents a sharp unimodal distribution but leans to the left (negative direction), indicating some extreme values or abnormal points. It is still close to normal distribution overall, but has a certain skewness due to asymmetric tail, and the data dispersion is high; X3 has two obvious peaks, two main data clusters, and large dispersion; X4 has an obvious sharp peak, close to uniform distribution, and small dispersion; X5 has a relatively gentle overall trend with a certain skewness, and medium dispersion; X6 presents a standard normal distribution curve, with data evenly distributed around the mean, good symmetry and stability; X7 shows a slight bimodal distribution trend, which is not obvious visually. Most data seem to cluster in a central area, and data on both sides gradually decrease; X8 shows strong skewness, that is, most data are concentrated on the left, and a long tail appears on the right, but the data still maintain certain continuity and regularity; X9 has a unimodal distribution with a certain skewness and large dispersion; X10 presents a sharp unimodal normal distribution, evenly distributed; X11 shows a standard normal distribution curve with good symmetry and stability; X12 is unimodal, close to uniform distribution, with small dispersion; X13 has three peaks, data are clearly divided into three parts, and data dispersion is large; X14 is unimodal with a certain skewness, and data dispersion is large; X15 presents a sharp unimodal normal distribution, close to uniform distribution, with small dispersion; X16 is unimodal, data are more concentrated, with small dispersion; X17 is unimodal, data are between 0–2, with large dispersion.

3) Correlation

Since data usually conform to normal distribution, Pearson correlation coefficient is used to measure the correlation between two variables.

The correlation results between input variables of expressway traffic accident risk feature analysis considering spatio-temporal effects are shown in Fig 3. Among them, red indicates a positive correlation between two variables, and blue indicates a negative correlation. It can be seen that the two variables with the highest positive correlation are X14 and X5, with a correlation coefficient of 0.96, indicating that the larger the rear vehicle speed difference, the larger the following distance. X17 and X4 have a high positive correlation, with a correlation coefficient of 0.91, indicating that the standard deviation of acceleration difference between front and rear vehicles increases with the increase of the standard deviation of rear vehicle acceleration. In addition, positive correlations include X16 and X3, X15 and X2, X17 and X2, X10 and X2, X9 and X1, X17 and X12, X15 and X10, X17 and X10; the negative correlation is X8 and X7, with a correlation coefficient of −0.77, indicating that the smaller the mean time to collision, the larger the standard deviation of time to collision, and the more unstable it is. Since the correlation between all parameters reaches the strong correlation threshold of 0.6, it indicates that the collinearity between variables is prominent, as sorted out in Table 8.

**Table 8:** Correlation indicators.

Correlation Indicators	Determined Indicators
X14、X5	X5
X17、X4	X4
X16、X3	X3
X15、17、10、X2	X2
X9、X1	X1
X17、X12	X12
X15、X17、X10	\
-X8、X7	X7

**Fig 3:**
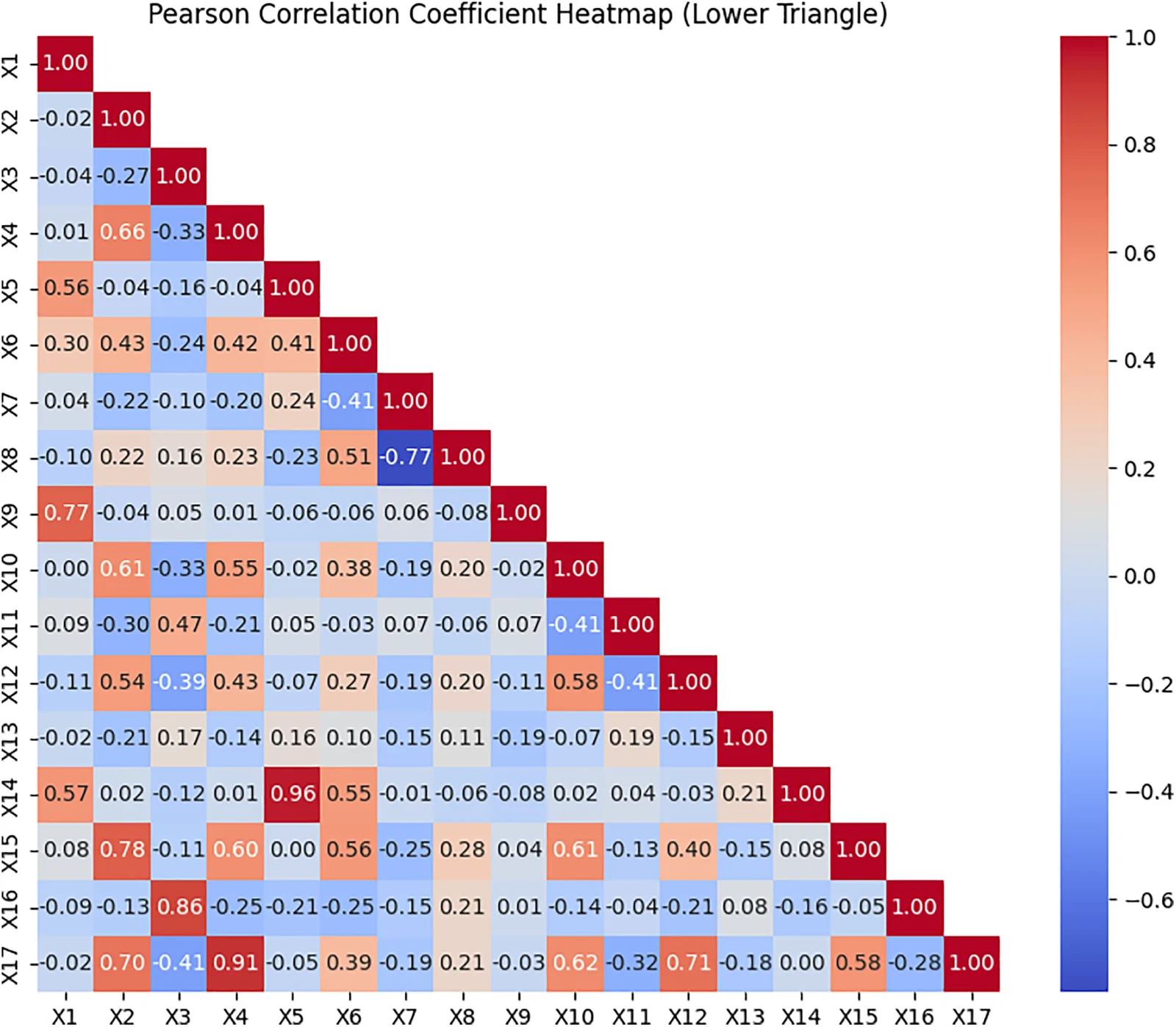
Feature correlation analysis.

Therefore, it is recommended to select indicators X1, X2, X3, X4, X5, X6, X7, X11, X12, X13, which have no obvious collinearity.

#### Traffic accident risk classification considering spatio-temporal effects.

Aiming at the challenge of data processing of multi-source heterogeneous traffic features, this section innovatively constructs a “feature dimensionality reduction-cluster recognition” two-level classification model. Firstly, features are compressed through Principal Component Analysis (PCA) to effectively solve the problem of variable collinearity; then, Fuzzy C-Means (FCM) algorithm is used to divide risks into three levels: serious, general and minor according to core indicators such as headway and time to collision. This classification model not only solves the problem of fuzzy risk boundaries in traditional methods, but also reveals the key causation mechanism of different risk levels through cluster center feature inversion.

PCA-based feature extraction is a data dimensionality reduction technology aimed at retaining key features, removing noise and unimportant information, thereby improving data processing efficiency. The main steps of PCA are as follows.

(1) Standardization

Calculate the mean$\overset{―}{x_{j}}$and standard deviation$S_{j}$ of each feature (total p features), the formulas are as follows:


$$\begin{array}{c} \begin{array}{c} \overset{―}{x_{j}}=\frac{\text{1}}{n}\sum_{i=\text{1}}^{n}\;x_{ij} \end{array} \end{array}$$ (4)

$$\begin{array}{c} \begin{array}{c} S_{j}=\sqrt{\frac{\sum_{i=\text{1}}^{n}\;{\left(x_{ij}-\overset{―}{x_{j}}\right)}^{\text{2}}}{n-\text{1}}} \end{array} \end{array}$$ (5)

Standardize each feature of each sample to obtain the standardized feature $X_{stand}$:


$$\begin{array}{c} \begin{array}{c} X_{ij}=\frac{x_{ij}-\overset{―}{x_{j}}}{S_{j}} \end{array} \end{array}$$ (6)

$$\begin{array}{c} \begin{array}{c} X_{\text{stand}}=[\begin{array}{cccc} X_{\text{11}} & X_{\text{12}} & \cdots & X_{\text{1}p}\\ X_{\text{21}} & X_{\text{22}} & \cdots & X_{\text{2}p}\\ \vdots & \vdots & \ddots & \vdots \\ X_{n\text{1}} & X_{n\text{2}} & \cdots & X_{np} \end{array}]=\left[X_{\text{1}},X_{\text{2}},\cdots ,X_{p}\right] \end{array} \end{array}$$ (7)

(2) Calculate Covariance Matrix

The covariance matrix is a table summarizing the correlation between all possible paired variables.


$$\begin{array}{c} \begin{array}{c} X_{ij}=\frac{x_{ij}-\overset{―}{x_{j}}}{S_{j}} \end{array} \end{array}$$ (8)

$$\begin{array}{c} \begin{array}{c} R=[\begin{array}{cccc} r_{\text{11}} & r_{\text{12}} & \cdots & r_{\text{1}p}\\ r_{\text{21}} & r_{\text{22}} & \cdots & r_{\text{2}p}\\ \vdots & \vdots & \ddots & \vdots \\ r_{p\text{1}} & r_{p\text{2}} & \cdots & r_{pp} \end{array}] \end{array} \end{array}$$ (9)

$$\begin{array}{c} \begin{array}{c} \begin{array}{cc} r_{ij} & =\frac{\text{1}}{n-\text{1}}\sum_{k=\text{1}}^{n}\;\left(X_{ki}-\overset{―}{X_{i}}\mathrm{\backslash rightleft}(X_{kj}-\overset{―}{X_{j}}\right)\\ & =\frac{\text{1}}{n-\text{1}}\sum_{k=\text{1}}^{n}\;X_{ki}X_{kj} \end{array} \end{array} \end{array}$$ (10)

(3) Calculate Eigenvalues and Eigenvectors

Calculate the eigenvalues of matrix R, arrange them in descending order, calculate the corresponding eigenvectors, and standardize them to make their length 1.

(4) Adaptability Test

Before applying Principal Component Analysis (PCA), the suitability of the data needs to be tested. To verify the correlation structure between variables, the sphericity test is first used to test the applicability of the multi-dimensional variable system. The test results show that the Bartlett statistic reaches 71087.8133 (corresponding p-value is much less than 0.05), indicating that there is a significant correlation between variables, meeting the prerequisite of principal component analysis; at the same time, the KMO value comprehensively exceeds the 0.5 threshold, and most variable combinations have a KMO value of more than 0.7, showing that the data has a good partial correlation structure. According to the Kaiser-Meyer-Olkin metric standard, when KMO > 0.5, it indicates that the data is suitable for principal component analysis. The test results of this study are significantly better than the critical value requirements, so PCA is carried out.

(5) Calculate Principal Component Contribution Rate and Cumulative Contribution Rate

The contribution rate of the i-th principal component is:


$$\begin{array}{c} \begin{array}{c} \frac{\lambda_{i}}{\sum_{k=\text{1}}^{p}\;\lambda_{k}} \end{array} \end{array}$$ (11)

The cumulative contribution rate of the first i principal components is:


$$\begin{array}{c} \begin{array}{c} \frac{\sum_{j=\text{1}}^{i}\;\lambda_{j}}{\sum_{k=\text{1}}^{p}\;\lambda_{k}} \end{array} \end{array}$$ (12)

(6) Select and Represent Principal Components

In PCA, the first m principal components (m ≤ p, p is the number of original variables) corresponding to eigenvalues with cumulative contribution rate exceeding 80% are usually selected. Fi represents the 𝕚-th principal component.


$$\begin{array}{c} \begin{array}{c} F_{i}=l_{i\text{1}}X_{\text{1}}+l_{i\text{2}}X_{\text{2}}+\cdots +l_{ip}X_{p},(i=\text{1},\text{2},\ldots ,pnonumber \end{array} \end{array}$$ (13)

For the 17-dimensional feature vector contained in the original dataset, this study implements principal component analysis to extract core information components. The eigenvalues and their variance contribution rates of each principal component are obtained through calculation. According to the dual screening criteria of eigenvalue greater than 1 and cumulative variance contribution rate exceeding 80%, the first 6 principal components are finally determined to be retained.

From the principal component analysis, PC1 is mainly contributed by X2, X4, X10, X17, PC2 is mainly contributed by X1, X5, X14, etc., and then the main parts are extracted according to the correlation analysis of each variable. PC1 is mainly composed of the standard deviation of rear vehicle speed, standard deviation of rear vehicle acceleration, and standard deviation of front vehicle acceleration. PC2 is mainly composed of the average speed of rear vehicle and average headway. PC3 is mainly composed of the average acceleration of rear vehicle and average time to collision. PC4 is mainly composed of the average speed of rear vehicle, average speed of front vehicle, and average lane change. PC5 is mainly composed of the average acceleration of rear vehicle, average time to collision, and average acceleration difference between two vehicles. PC6 is mainly composed of the average acceleration of rear vehicle, average time to collision, and average acceleration difference between two vehicles.

In traffic accident risk classification, FCM is a very effective tool due to its advantages in handling fuzziness and uncertainty, providing flexible risk division, and adapting to complex data distribution. Based on the FCM clustering algorithm, this study uses the sklearn-fuzzy library of Python for pattern recognition of traffic flow data. By minimizing the weighted intra-class distance objective function, setting the fuzzy index m = 2 clustering number C = 3, and maximum iteration number max_iter = 100, the accurate division of mixed traffic flow data is realized. The algorithm iteratively optimizes the membership matrix and cluster center coordinates, and finally converges to a stable solution. The visualization result (Fig 4) clearly shows the cluster boundaries in the three-dimensional feature space, and the cluster center coordinates recorded in Table 9 further verify the effectiveness of the algorithm.

**Table 9:** Cluster center table.

Cluster Center	PC1	PC2	PC3	PC4	PC5	PC6
**1**	−0.094	1.740	0.076	−0.365	−0.015	−0.094
**2**	9.760	−0.375	−1.608	0.295	0.866	9.760
**3**	−0.448	−1.003	0.038	0.199	−0.036	−0.448

**Fig 4:**
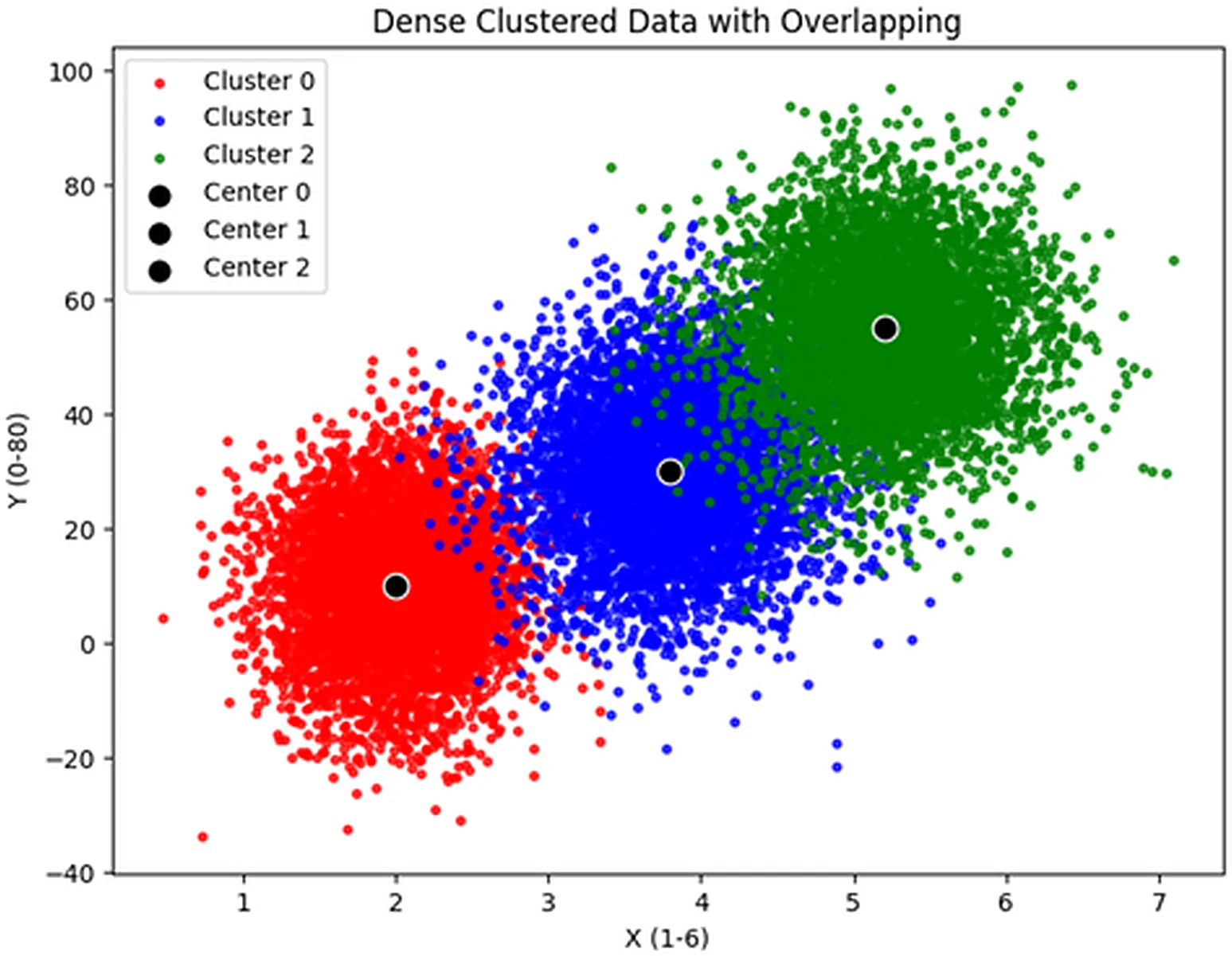
Clustering results plot.

According to the cluster center table, the main load of each principal component is used to identify accident risk categories, check the position of each cluster in the principal component space, and the original variables corresponding to these positions. Determine that each original variable is significantly correlated with the clustering result, and then obtain which factors drive the clustering result according to their correlation. According to the analysis of clustering results based on the proportion of main components of PC1 to PC6 (Table 9), they are sorted out in Table 10.

**Table 10:** Clustering results of principal components corresponding to major original variables.

Cluster Center	Standard Deviation of Rear Vehicle Speed	Average Headway	Average Time to Collision	Average Speed of Front Vehicle	Average Acceleration of Front Vehicle	Average Acceleration Difference Between Two Vehicles
**1**	0.16	24.66	2.11	26.42	0.04	−0.04
**2**	0.11	43.00	3.56	25.42	0.09	−0.04
**3**	0.17	11.43	0.98	26.86	0.12	−0.11

According to the clustering result analysis, cluster center 3 has the lowest average time to collision (0.98 s), the lowest average headway (11.43 m), and the largest acceleration difference between two vehicles (−0.11), indicating the most dangerous driving state, so this category is defined as serious accident risk. Cluster center 1 has intermediate average time to collision (2.11 s) and average headway (24.66 m), defined as general accident risk. Cluster center 2 has the highest average time to collision (3.56 s) and average headway (43.00 m), defined as minor accident risk. This classification provides clear target variables for the subsequent CNN-BiLSTM supervised learning model.

The accident risk level is essentially a latent state that cannot be observed directly, and naturalistic trajectory data do not provide ready-made risk labels. This study therefore adopts a two-stage “feature dimensionality reduction-cluster recognition” labeling strategy: PCA decorrelates the 17-dimensional traffic-interaction features and retains six principal components with a cumulative contribution rate of 83.449%, and FCM then partitions the reduced feature space into serious, general and minor risk states. Compared with direct TTC-threshold labeling, K-Means hard clustering, or manual expert labeling, the PCA-FCM strategy has four advantages. First, it is data-driven and does not rely on a single subjective risk threshold. Second, it characterizes risk through the joint distribution of multiple indicators rather than a single safety measure. Third, fuzzy membership appropriately handles the ambiguous and overlapping boundaries between risk levels. Fourth, cluster-center inversion maps each risk cluster back to the original traffic-interaction features (e.g., TTC, headway and acceleration difference), making the generated labels physically interpretable. According to the FCM clustering output, each sample is assigned to one of the three risk categories, which provides the target variable for the subsequent CNN-BiLSTM supervised learning model.

### Construction and optimization of prediction model

Traditional single neural network models have obvious limitations in processing complex spatio-temporal data. This section proposes two improved architectures: CNN-LSTM and CNN-BiLSTM, which realize collaborative processing of spatio-temporal information through a deep feature fusion mechanism. Based on the CNN module to extract spatial local features of traffic data and the BiLSTM module to capture forward and backward long-term dependencies of time series, an end-to-end traffic accident risk prediction framework is constructed. This model can not only effectively handle the nonlinear spatio-temporal relationship in traffic data, but also adapt to risk prediction requirements under different road environments and traffic conditions.

#### CNN-LSTM model.

In the prediction of spatio-temporal effects of traffic accidents, to model nonlinear features more efficiently, the CNN-LSTM network architecture is adopted. The CNN-LSTM network combines the advantages of CNN in spatial feature extraction and LSTM in time series modeling, can effectively handle complex nonlinear data, and is suitable for the prediction of spatio-temporal effects of traffic accidents, as shown in Fig 5.

**Fig 5:**
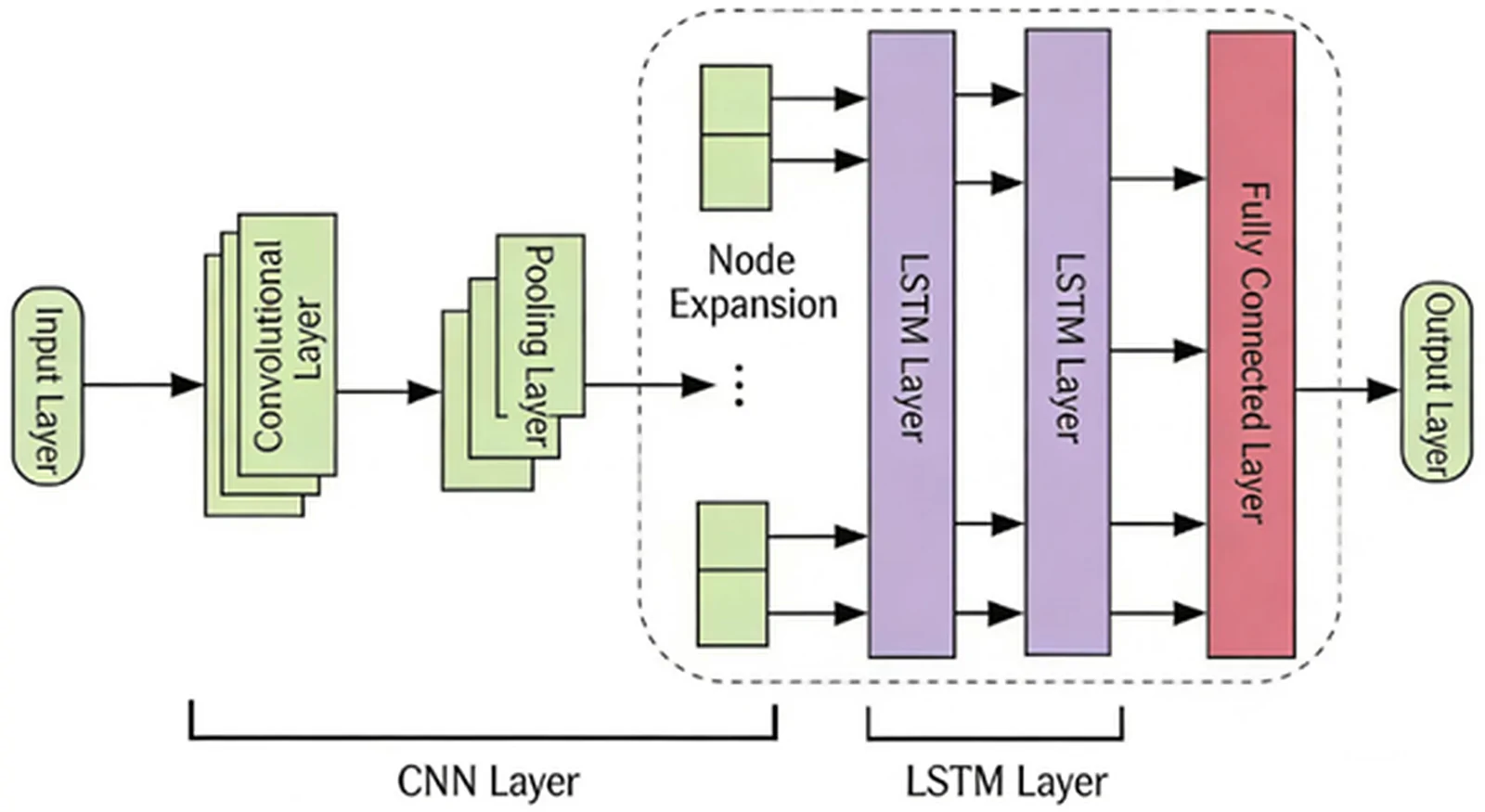
CNN-LSTM network structure.

The CNN-LSTM model receives multi-dimensional original data through the input layer, extracts local spatial features through the convolutional layer, reduces dimensions through the pooling layer, captures long-term dependencies of time series through the LSTM layer, finally maps spatio-temporal fusion features to the target space through the fully connected layer, and generates prediction results through the output layer. By combining the spatial feature extraction capability of CNN and the time-dependent modeling capability of LSTM, the CNN-LSTM model can effectively handle complex sequence data tasks. Its core structure includes convolutional layer, LSTM layer and fully connected layer, which is suitable for time series prediction. The model performance can be further improved by adjusting model parameters and optimizing the training process.

#### CNN-BiLSTM model.

The CNN-BiLSTM model can handle both spatial and temporal features of data by combining CNN and BiLSTM. The CNN part is responsible for extracting spatial features of input data, while the BiLSTM part processes time series data and captures long-term sequence dependencies. The CNN-BiLSTM model receives preprocessed numerical text data through the input layer, extracts local features through the convolutional layer, reduces dimensions through the pooling layer, captures the forward and backward context information of the sequence through the bidirectional LSTM layer, finally summarizes features and maps to the target space through the fully connected layer, and generates category probability or continuous value prediction results through the output layer according to the task type.

In the CNN-BiLSTM model, CNN extracts local features of data through convolution and pooling operations to generate feature maps, then flattens these feature maps into one-dimensional vectors and inputs them into BiLSTM, as shown in Fig 6.

**Fig 6:**
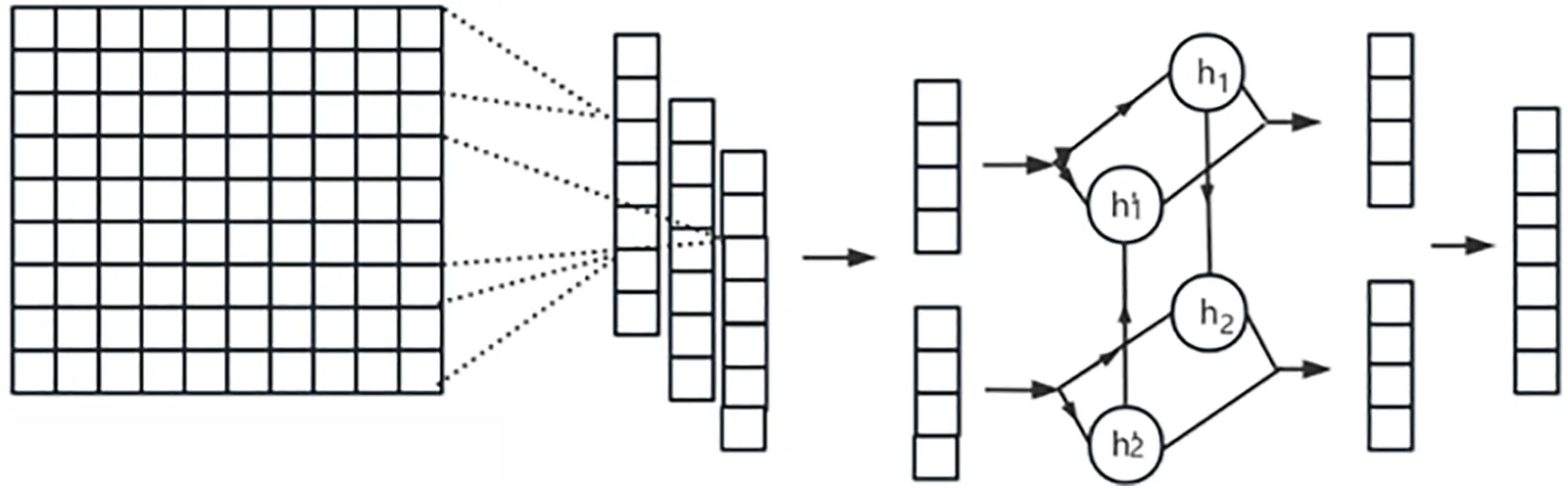
Flow chart of CNN-BiLSTM network structure.

Compared with the CNN-LSTM model, the main adjustment of the CNN-BiLSTM model is that the unidirectional LSTM layer is replaced by a bidirectional LSTM layer. The bidirectional structure processes the traffic parameter sequence from both the forward and backward directions and merges the two directional outputs with the concat mode, so that the model can simultaneously capture the temporal dependencies before and after a risk event. This adjustment is motivated by the expressway accident-risk task: accident risk is influenced by both the evolution of traffic flow before the accident and the change of traffic state after the accident, while unidirectional temporal modeling can only exploit historical information. Therefore, the bidirectional design is not an arbitrary structural replacement but a scenario-driven improvement for capturing the spatio-temporal interaction of expressway traffic accident risk.

### Model application and evaluation

This chapter focuses on the empirical research of the expressway accident risk prediction model, and verifies the model performance through systematic experimental design. Based on the HighD dataset, spatio-temporal feature engineering is constructed, a hybrid deep learning architecture is adopted, combined with grid search and time series cross-validation optimization strategies, and a comprehensive evaluation is carried out from three dimensions: prediction accuracy, computational efficiency and classification performance.

#### Model structure optimization and process.

Model performance optimization is a key link to ensure the reliability of prediction results. This section elaborates on the network hyperparameter tuning strategy, including sensitivity analysis of convolution kernel size, determination method of LSTM unit number and optimization process of dropout ratio. Through designing an end-to-end model training process, the whole chain optimization from data preprocessing to prediction output is realized.

The structure optimization of CNN-LSTM model and CNN-BiLSTM model is the process of improving model performance by adjusting model architecture, hyperparameters and training strategies. The detailed methods for structure optimization of CNN-LSTM model and CNN-BiLSTM model are as follows.

(1) Model structure optimization methods, including adjusting the CNN layer by increasing the number of layers to extract more complex features, increasing the number of filters to capture more feature patterns, using dilated convolution to expand the receptive field to capture long-distance dependencies; adjusting the LSTM layer by increasing the number of units to enhance memory ability, adopting bidirectional LSTM to model forward and backward temporal dependencies at the same time, stacking multi-layer LSTM to extract complex time features; adding regularization layers such as Dropout to randomly discard neurons to prevent overfitting, or adding weight penalty items to the loss function to limit model complexity; and adjusting the fully connected layer by increasing or decreasing the number of layers and neurons to balance model expression ability and generalization performance.
(2) Hyperparameter optimization, including using LearningRateScheduler to dynamically adjust the learning rate, using a smaller batch size to improve training stability, using early stopping to prevent overfitting, and trying different optimizers such as Adam, RMSprop, SGD.
(3) Enhance input data to increase data diversity. Standardize or normalize input data to accelerate model convergence. Adjust the sliding window size to capture time dependencies of different lengths.

Aiming at the problem of complex nonlinear time series prediction, this study proposes a hybrid modeling framework integrating CNN and LSTM. By systematically deconstructing the local feature extraction and long-term dependency modeling of time series, this study constructs an end-to-end depth prediction system. The specific implementation path covers the following key technical links, and the process of CNN-LSTM model is shown in Fig 7.

**Fig 7:**
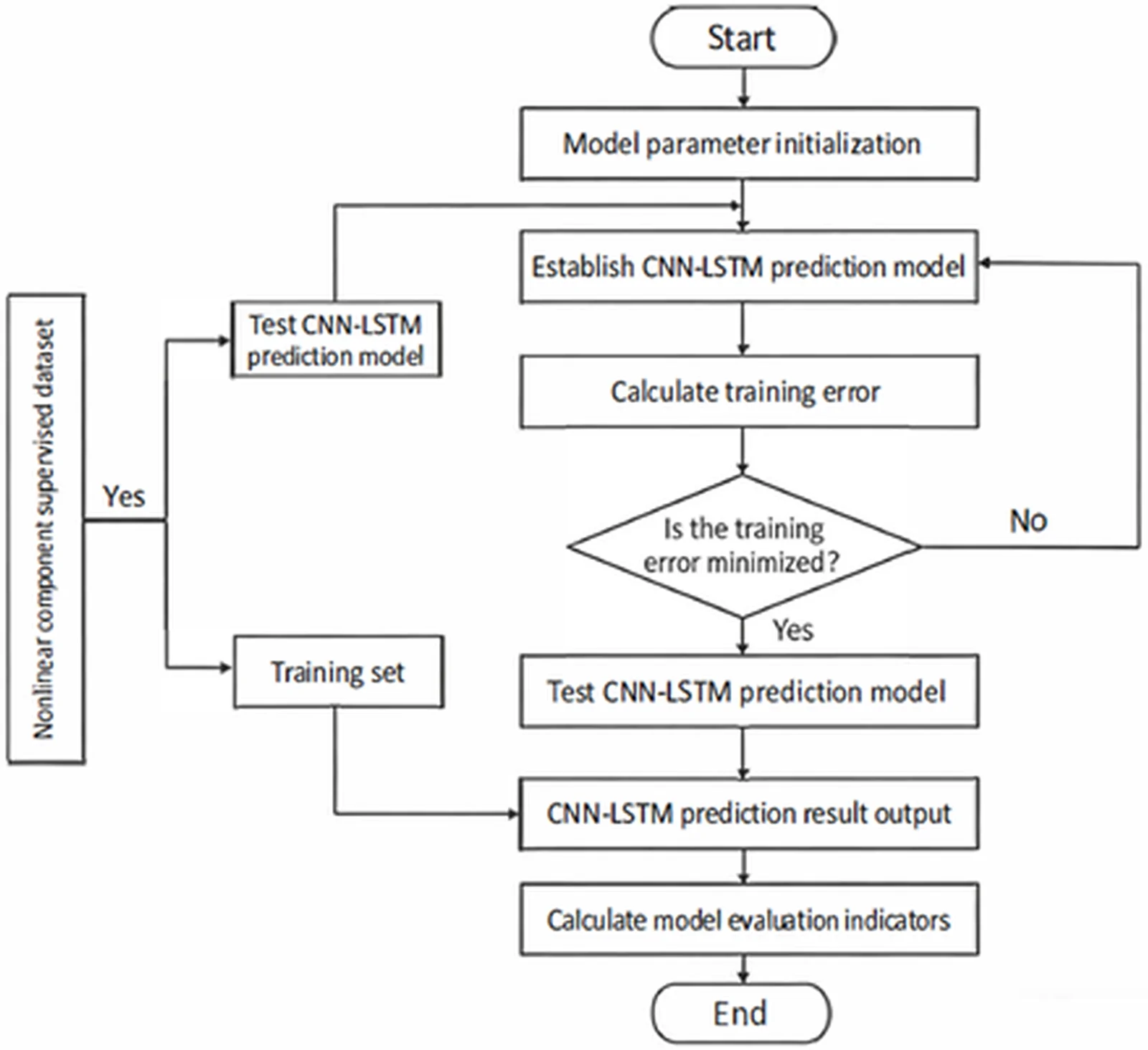
Flow chart of CNN-LSTM network structure.

Aiming at the coupling challenge of local feature extraction and bidirectional temporal dependency modeling in complex nonlinear time series prediction, this study proposes a prediction framework based on deep spatio-temporal feature fusion. By systematically constructing a feature representation space under the supervised learning paradigm, the local pattern recognition ability of CNN and the global temporal modeling advantage of Bidirectional Long Short-Term Memory (BiLSTM) are organically combined to form a collaborative prediction mechanism of “spatial feature extraction-temporal dependency capture.” The specific implementation path covers the following key technical links, and the process of CNN-BiLSTM model is shown in Fig 8.

**Fig 8:**
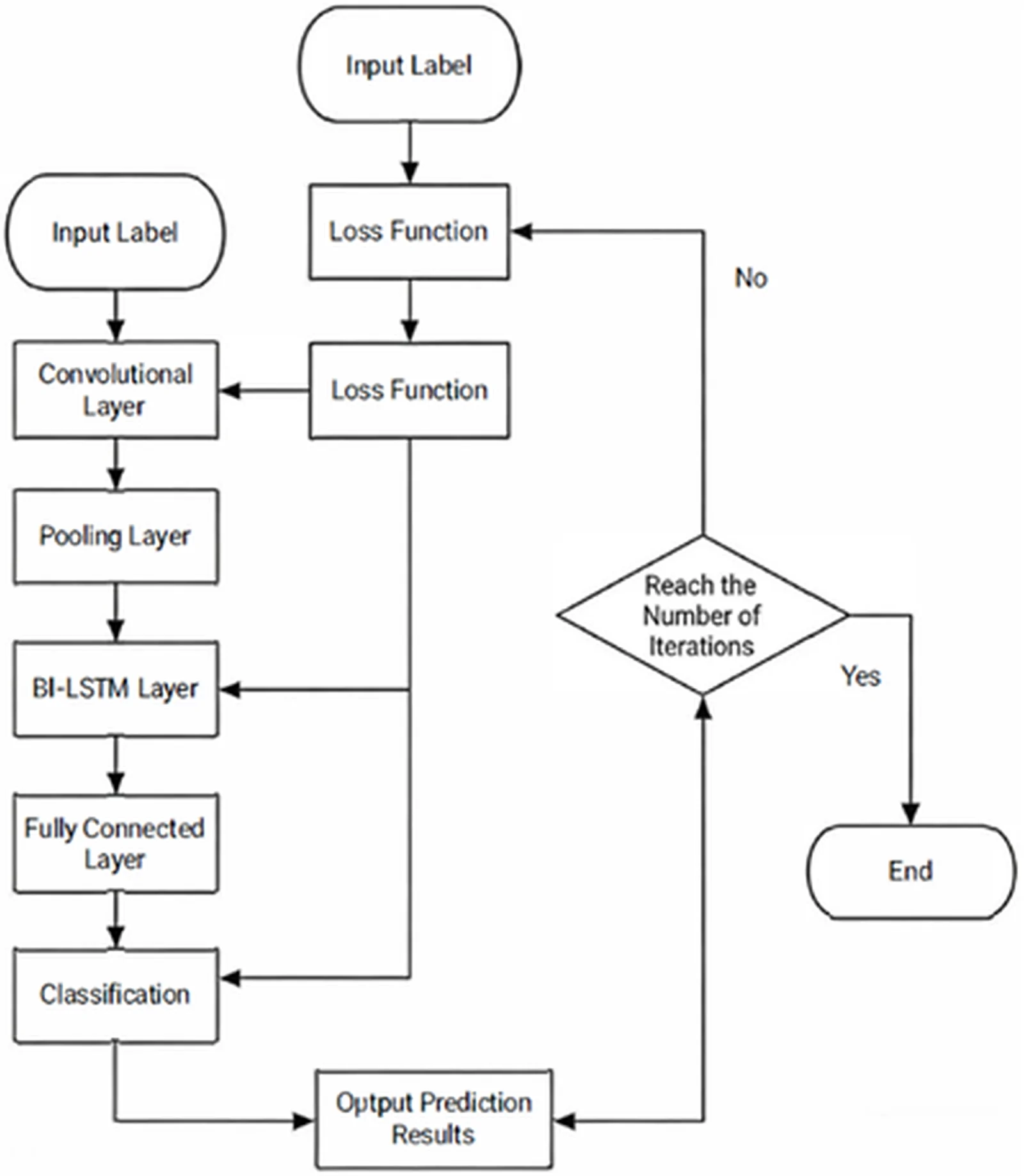
Flow chart of CNN-BiLSTM network structure.

#### Data preparation.

Based on the HighD expressway vehicle trajectory dataset, this section ensures data quality through a strict preprocessing process. Regarding the time effect of accident risk, this paper mainly analyzes two scenarios: the duration pattern of accidents and the time period during which the accident risk is severe. The total distribution of traffic accident risk frames (i.e., data when TTC values are less than 4 seconds) is shown in Fig 9.

**Fig 9:**
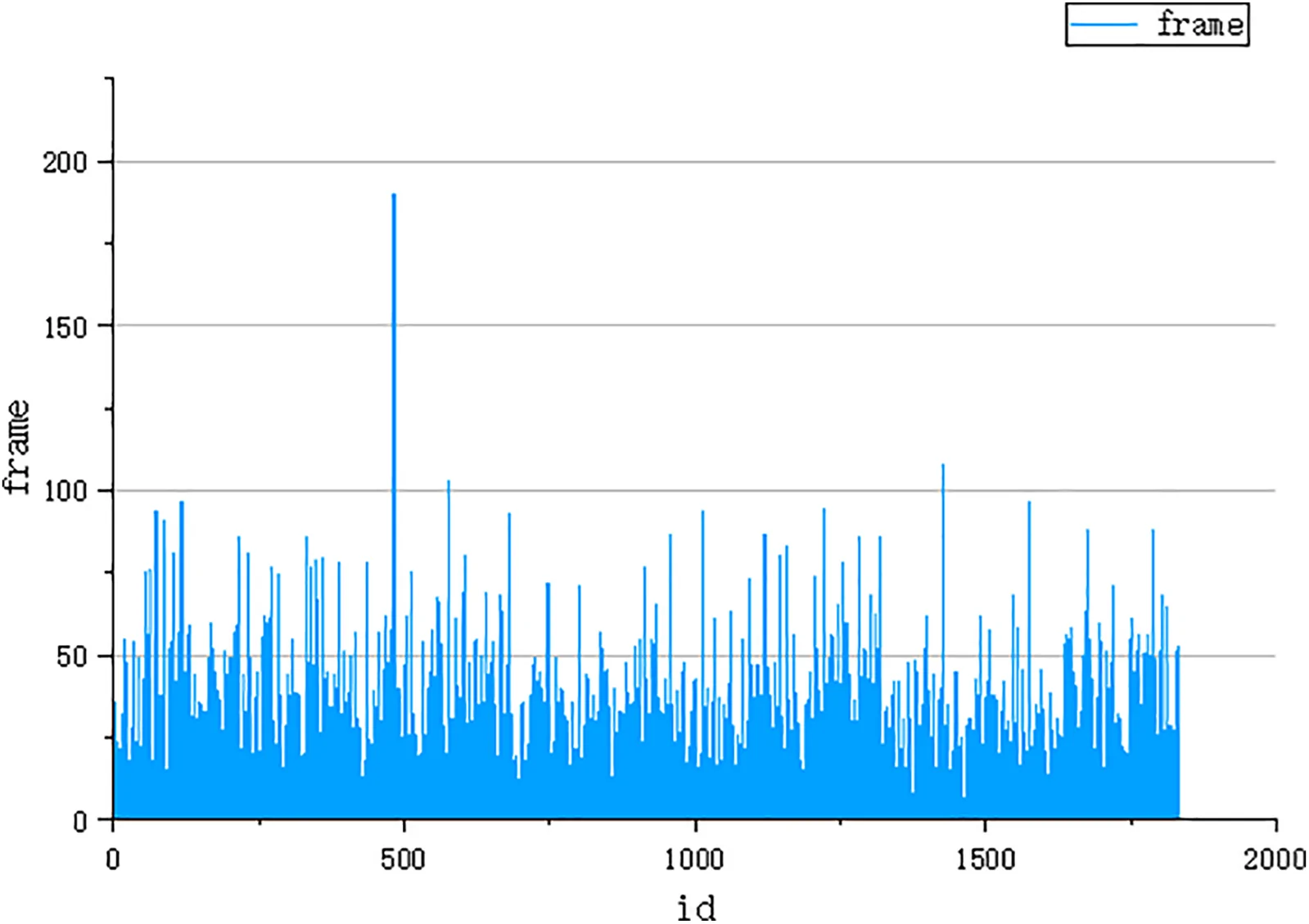
Frame size distribution of at-risk vehicles.

Based on the frame count distribution of each risk vehicle, data are generally distributed within 0–100 frames, with 0–50 frames being denser. The distribution of 0–100 frames is further described and analyzed based on the proportion of their numbers and the rate of change, as shown in Table 11.

**Table 11:** Traffic accident data from 0 to 100 frames.

Distribution	Frame count	Quantity	Total	Percentage of the market
1 ~ 25 frame	30170	1192	1833	65.03%
26 ~ 50 frame	9285	489	1833	26.68%
51 ~ 75 frame	1991	119	1833	6.49%
76 ~ 100 frame	395	29	1833	1.58%
101 ~ 125 frame	36	2	1833	0.22%

According to Table 11, traffic accidents generally occur within the first 1–50 frames, which is equivalent to ending within 0–2 seconds at the time of the accident. 65.03% of dangerous events occur within 0–1 s, 26.68% within 1–2 s, and over 91% are concentrated within the 0–4 s interval, confirming that this window captures the critical accident evolution phase while excluding unrelated noise.

The lower bound of the pre-accident feature window (4 s) aligns with the TTC risk threshold to ensure temporal continuity between feature and label windows, while the upper bound (10 s) is determined based on car-following stability analysis from related studies. Furthermore, to prevent information leakage, the dataset is partitioned strictly by vehicle accident event IDs, ensuring that all temporal segments of the same event remain exclusively in either the training or test set. This is complemented by Time Series Split cross-validation during hyperparameter optimization, which guarantees that the test set chronologically follows the training set. Ensure data integrity and accuracy, and clean and correct missing or abnormal data as necessary.

To evaluate the model prediction performance, it is necessary to compare the predicted value with the actual value. In this experiment, the dataset is divided into two parts: 127,320 pieces of data are used as the training set for model training; 31,830 pieces of data are used as the test set for verifying the model prediction effect. The specific division is shown in Table 12.

**Table 12:** Dataset division.

Dataset	Time Period	Training Set	Test Set
Before Accident	4s < TTC < 10s	97167	24292
At Accident Time	0s < TTC < 4s	30153	7538
Total	159150	127320	31830

#### Basic model parameter optimization.

In terms of parameter optimization, a hybrid parameter tuning strategy based on grid search and time series cross-validation is proposed. Independently optimize parameters of LSTM and CNN basic models to determine the optimal initial configuration; then, for CNN-LSTM and CNN-BiLSTM hybrid models, determine the optimal hyperparameter combination through multiple rounds of cross-validation.

When constructing the LSTM model in this paper, it is necessary to preset the activation function, batch-size, epoch, parameters of Adam optimizer (learning rate lr, parameters β1 and β2), training fitting error threshold ε, as well as the number of network layers and neurons. Referring to relevant literature, the parameter settings are shown in Table 13.

**Table 13:** Parameters of the LSTM base model.

Parameter	Batch-size	epoch	lr	$\beta_{1}$	$\beta_{2}$	ε
Value	8	20	0.001	0.9	0.999	10^−7^

When training the CNN model, the following basic parameters usually need to be set. These parameters can be adjusted based on experience, task requirements and related research. The following is a common CNN basic parameter setting, as shown in Table 14.

**Table 14:** Parameters of the CNN base model.

Parameter	Batch-size	epoch	lr	$\beta_{1}$	$\beta_{2}$	ε
Value	32	50	0.001	0.9	0.999	10^−8^

#### Improved model parameter optimization.

In this study, the hyperparameters of the neural network model are preset first, and then the grid search algorithm is used to comprehensively search the hyperparameter combinations to screen out multiple groups of better parameter configurations. Then, the Time Series Split method in scikit-learn is used for cross-validation. This method gradually expands the training set in chronological order, while ensuring that the test set can always be located after the training set to conform to the characteristics of time series data. The model performance within each cross-validation fold is evaluated, and the optimal configuration of each hyperparameter is selected according to the average validation performance across different folds. The independent test dataset is not involved in the hyperparameter optimization process and is only used for the final evaluation of model generalization performance.

This optimization procedure reduces the dependence of the final model configuration on a single manually selected parameter setting. The candidate hyperparameters are searched within predefined ranges, including kernel size, number of filters, pooling size, stride, batch size, dropout rate, and weight initialization method, as summarized in Table 15. The final parameter configuration is selected based on the cross-validation results obtained from the training data, thereby providing a systematic basis for parameter selection and reducing the potential influence of individual hyperparameter choices on model performance.

**Table 15:** Preset model parameters.

Parameter	Parameter Value
Kernel size	[3,5,6]
filters	[16,32,64]
Pool size	[2,3,4,5]
Lstm_output_size	32
strides	[1,2,3,4]
Batch size	[8,16,32,64]
Adam optimization	Adaptive
Dropout rate	[0.2,0.4,0.5,0.6,0.8,1.0]
Weight initialization method	[Normal, uniform]

The candidate ranges of the main hyperparameters investigated in the grid search are summarized in Table 15. These ranges were selected based on preliminary experiments and related studies to balance model complexity and prediction performance.

(1) NN-LSTM Model

After cross-validation and network search algorithm, the optimal parameter settings of the model are obtained, and the specific results are shown in Table 16. These parameters are determined through multiple iterations of grid search combined with time-series cross-validation on the training dataset, aiming to obtain the best generalization performance rather than maximizing training accuracy.

**Table 16:** CNN-LSTM Model parameter values.

Parameter	Parameter Value
Kernel size	(3,3)
filters	64
Units	128
Pool size	2
Lstm_output_size	32
strides	1
Batch size	8
Adam optimization	Adaptive
Dropout rate	0.4
Weight initialization method	uniform

(2) CNN-BiLSTM Model

When using the CNN-BiLSTM hybrid model for time series modeling, the hyperparameters of the model are systematically optimized through cross-validation and grid search algorithm, and the optimal hyperparameter combination is finally determined. The details are shown in Table 17.

**Table 17:** CNN-BiLSTM Model parameter values.

Parameter	Parameter Value
Kernel size	(3,3)
filters	64
Units	128
Pool size	2
Lstm_output_size	32
strides	1
Batch size	8
Adam optimization	Adaptive
Dropout rate	0.5
Weight initialization method	uniform
Bidirectional LSTM merging mode	concat

#### Evaluation method.

To conduct in-depth analysis and compare model performance, four evaluation indicators are adopted to evaluate the prediction effect of each model: Mean Squared Error (MSE), Root Mean Squared Error (RMSE), Mean Absolute Error (MAE) and Mean Absolute Percentage Error (MAPE). The larger the values of these four error indicators, the more significant the difference between the predicted value and the actual value, and the worse the prediction accuracy of the model. By analyzing the deviation between the quantitative model prediction results and the actual observation results, the comparative analysis of the fitted value and the true value is carried out, so as to understand the accuracy, reliability and applicability of the model.

To further intuitively compare the performance differences among models, Fig 10 presents a comparison between model fit values and true values. Visualization of model fitting performance Visualization Chart It can be seen that the CNN-LSTM model exhibits significant deviations during data reconstruction, especially during periods with significant sequence fluctuations, where the fitting curve deviates markedly from true values, indicating that this hybrid architecture has failed to effectively integrate convolutional features and temporal dependence in this experimental scenario.

**Fig 10:**
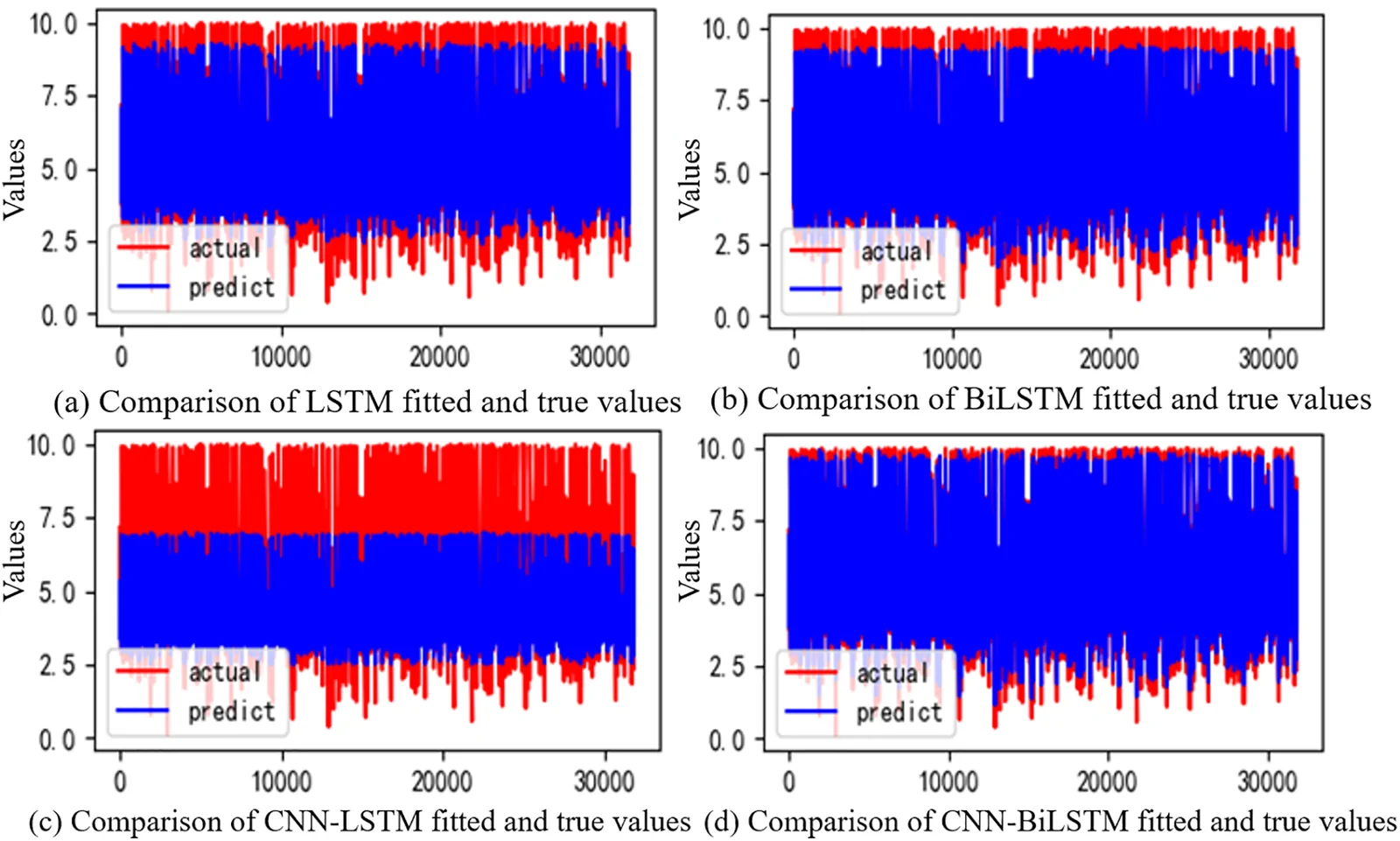
Comparison between the fitted value and the true value of the model. (a) Comparison of LSTM fitted and true values. (b) Comparison of BiLSTM fitted and true values. (c) Comparison of CNN-LSTM fitted and true values. (d) Comparison of CNN-BiLSTM fitted and true values.

## Results

### Comparative analysis of prediction results

It can be seen from the model fitting effect visualization chart that the CNN-LSTM model shows obvious deviation in the data reconstruction process, especially the fitting curve deviates significantly from the true value during the period of severe sequence fluctuation, indicating that this hybrid architecture fails to effectively integrate convolution features and temporal dependence in this experimental scenario. As shown in Fig 11, the blue LSTM, red BiLSTM and orange CNN-BiLSTM models have good fitting effects. The running time and error size of each model are evaluated and analyzed as follows.

**Fig 11:**
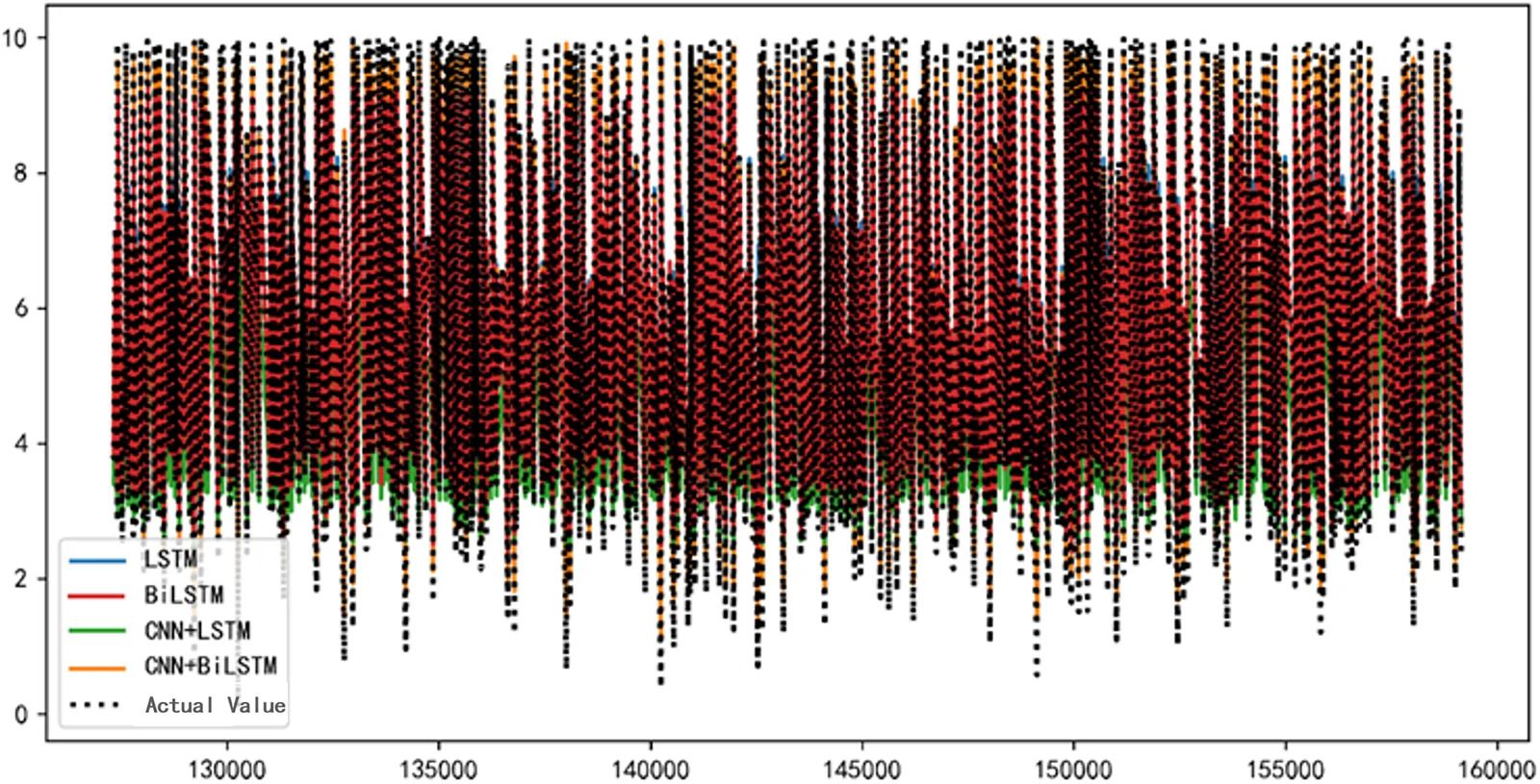
Comparative analysis of the models.

According to Fig 12, the BiLSTM model has the longest running time, the CNN-BiLSTM model has moderate running time, and the LSTM model has the shortest running time. Although the CNN-BiLSTM model introduces additional convolutional and bidirectional recurrent components compared with the single LSTM architecture, the running-time comparison in Fig 12 shows that its computational cost remains moderate among the evaluated models. This indicates that the additional feature extraction and bidirectional temporal modeling do not result in a disproportionate increase in computational cost. Considering its lower prediction errors and moderate running time, the CNN-BiLSTM model provides a reasonable trade-off between prediction accuracy and computational efficiency.

**Fig 12:**
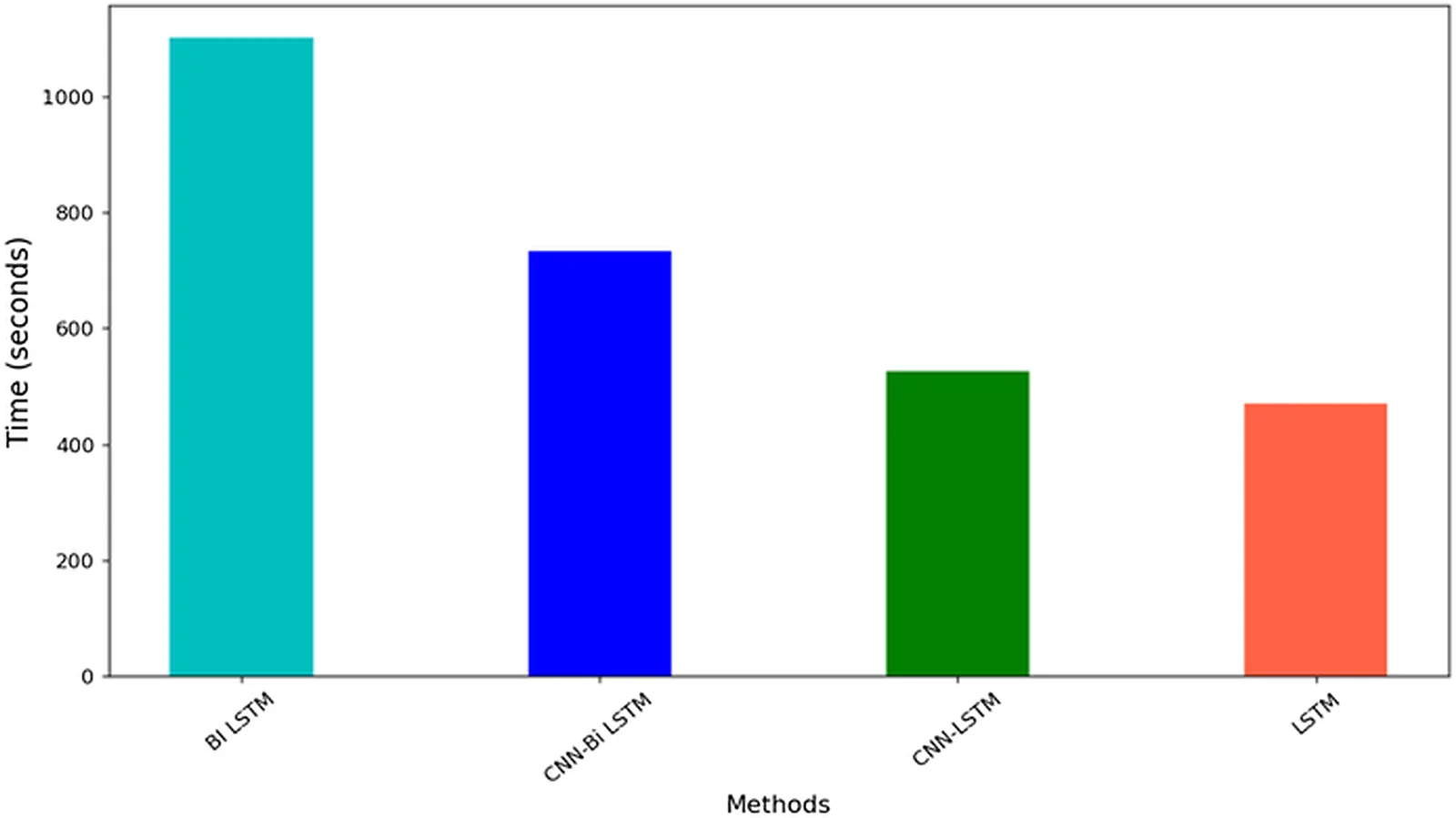
Comparison of running times.

Recent advanced models provide complementary advantages. Graph neural networks (GNNs) explicitly model spatial dependencies over road networks or vehicle interaction graphs and are well suited to regional or network-level traffic flow prediction. Spatio-temporal attention models adaptively select important time steps and spatial locations and can capture long-range temporal dependencies and multi-source feature interactions. Transformer-based spatio-temporal models further enable global parallel modeling of temporal dependencies but generally require more data and computational resources. For the present vehicle-level accident-risk task, the input consists of 17 traffic-interaction features extracted from HighD vehicle trajectories, and the risk evolution is concentrated in the short 0–4 s accident window and 4–10 s pre-accident window. This is a localized spatio-temporal pattern rather than a network-level topological pattern. GNNs require explicit graph construction or road-network topology that the HighD trajectory data do not directly provide, while attention and Transformer models increase parameter scale and computational cost without a clear matching advantage for this short-window vehicle interaction task. In contrast, CNN extracts local spatial features and BiLSTM captures forward and backward temporal dependencies, which matches the short-term vehicle-interaction risk task.

As shown in Fig 13, through error comparison, the CNN-BiLSTM model has MSE of 0.36, RMSE of 0.60, MAE of 0.20, and MAPE of 0.04, all of which are lower than other models. The model has the best accuracy, and combined with its moderate running time, this model is selected as the optimal one.

**Fig 13:**
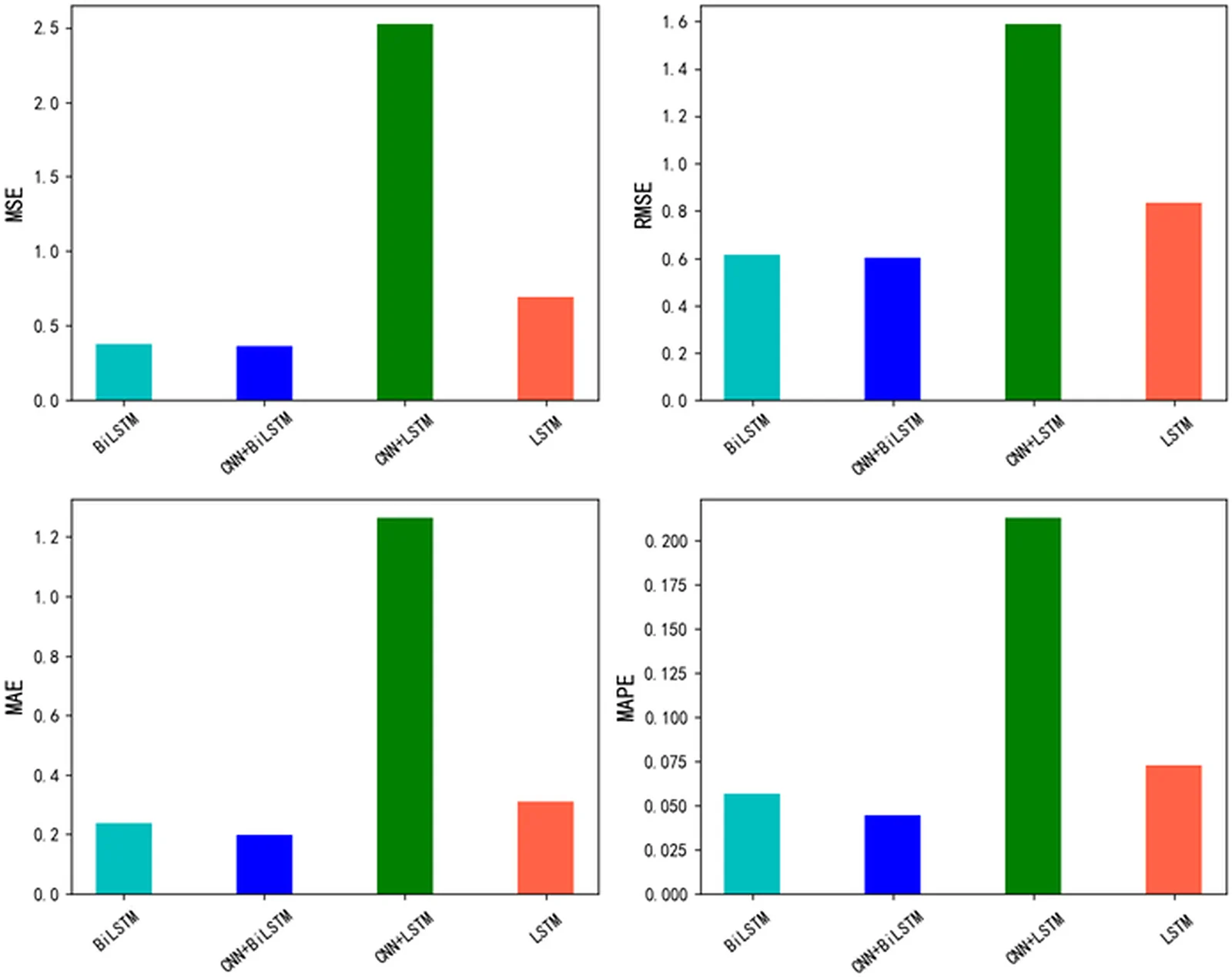
Error comparison and analysis.

### Classification analysis of prediction results

From the model comparison, the CNN-BiLSTM model is the optimal one. Using the test set data, the predicted value of this model is compared with the actual value. Fig 14 is comparison between the predicted and actual values.

According to the comparison Fig 14, the fitting effect of the predicted value is good. Therefore, it is used for classification prediction. According to the traffic accident risk classification, there are 3 types of accidents in total, and the prediction is divided into 4 cases, adding the category of no accident. According to the traffic accident risk classification standard, the prediction fitting is carried out, the confusion matrix is constructed, and the prediction results are obtained by using evaluation indicators such as accuracy, recall rate and F1 score. The specific fitting situation is shown in Fig 15.

**Fig 14:**
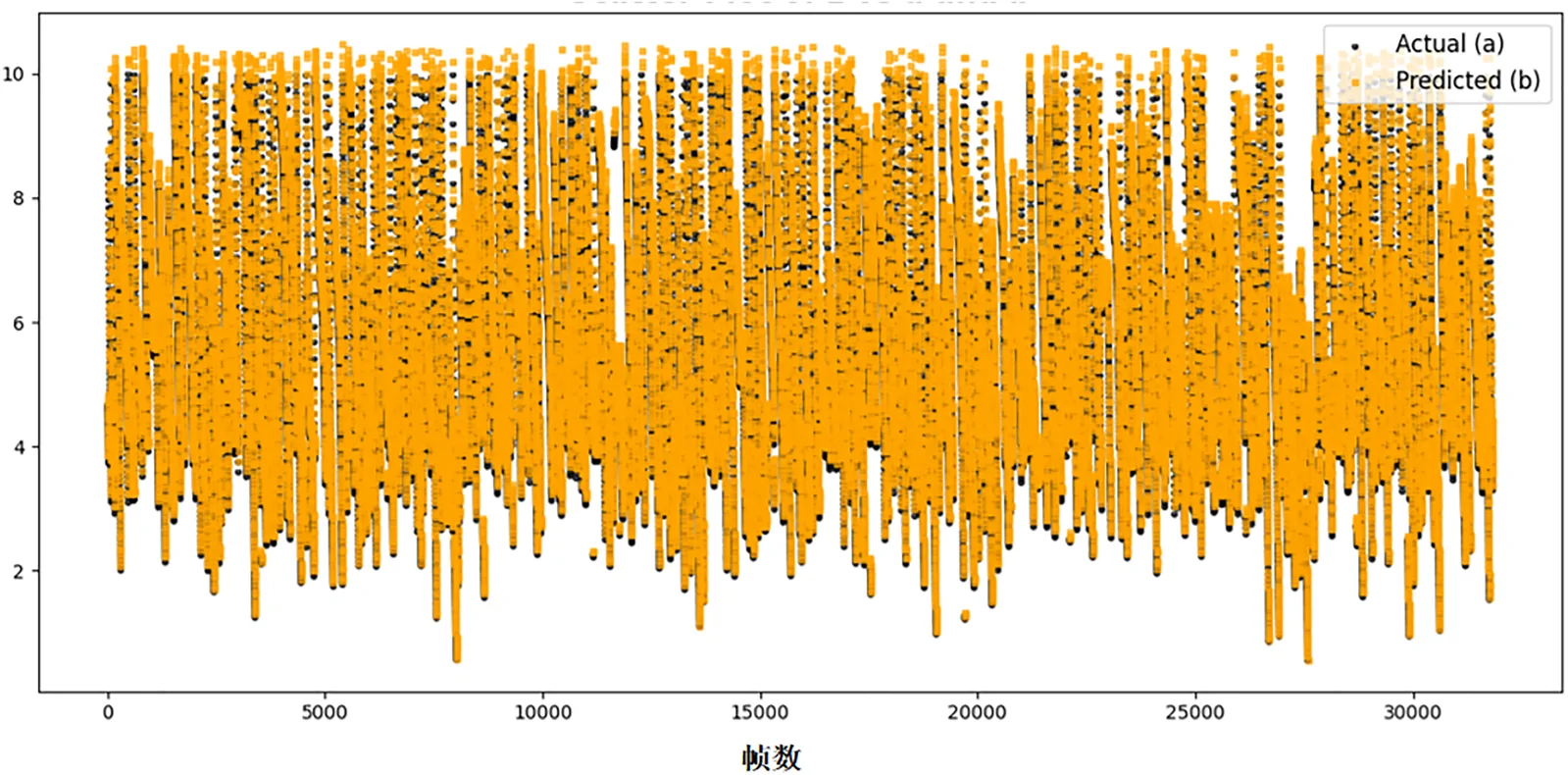
Comparison between the predicted and actual values.

**Fig 15:**
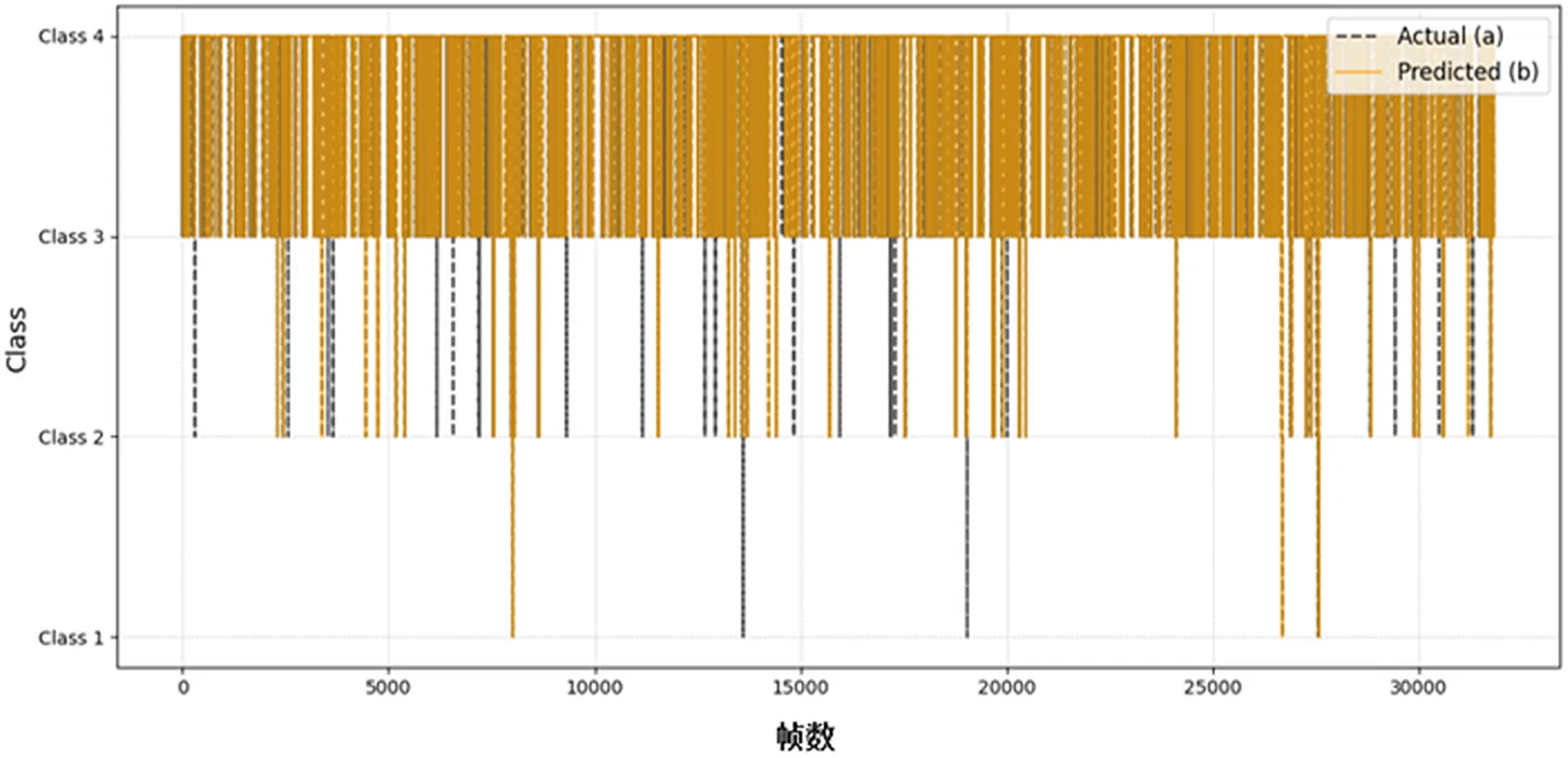
Fitting traffic accident risk classification chart.

As shown in the confusion matrix in Fig 16 and the traffic accident risk classification evaluation results in Table 18, all four classes achieve accuracy rates of approximately 0.90, demonstrating the model’s good capability in distinguishing different traffic accident risk categories. The model can effectively identify serious accidents, general accidents, minor accidents, and non-accident conditions. Overall, these results indicate that the proposed CNN-BiLSTM model provides reliable performance for traffic accident risk classification. The resulting risk predictions can also provide useful information for traffic information systems, supporting traffic risk monitoring and safety management.

**Table 18:** Fitting the traffic accident risk classification assessment table.

	precision	recall	f1-score	support
Class1	0.90	0.75	0.82	24
Class2	0.92	0.74	0.82	466
Class3	0.92	0.80	0.85	8227
Class4	0.93	0.98	0.96	23114

**Fig 16:**
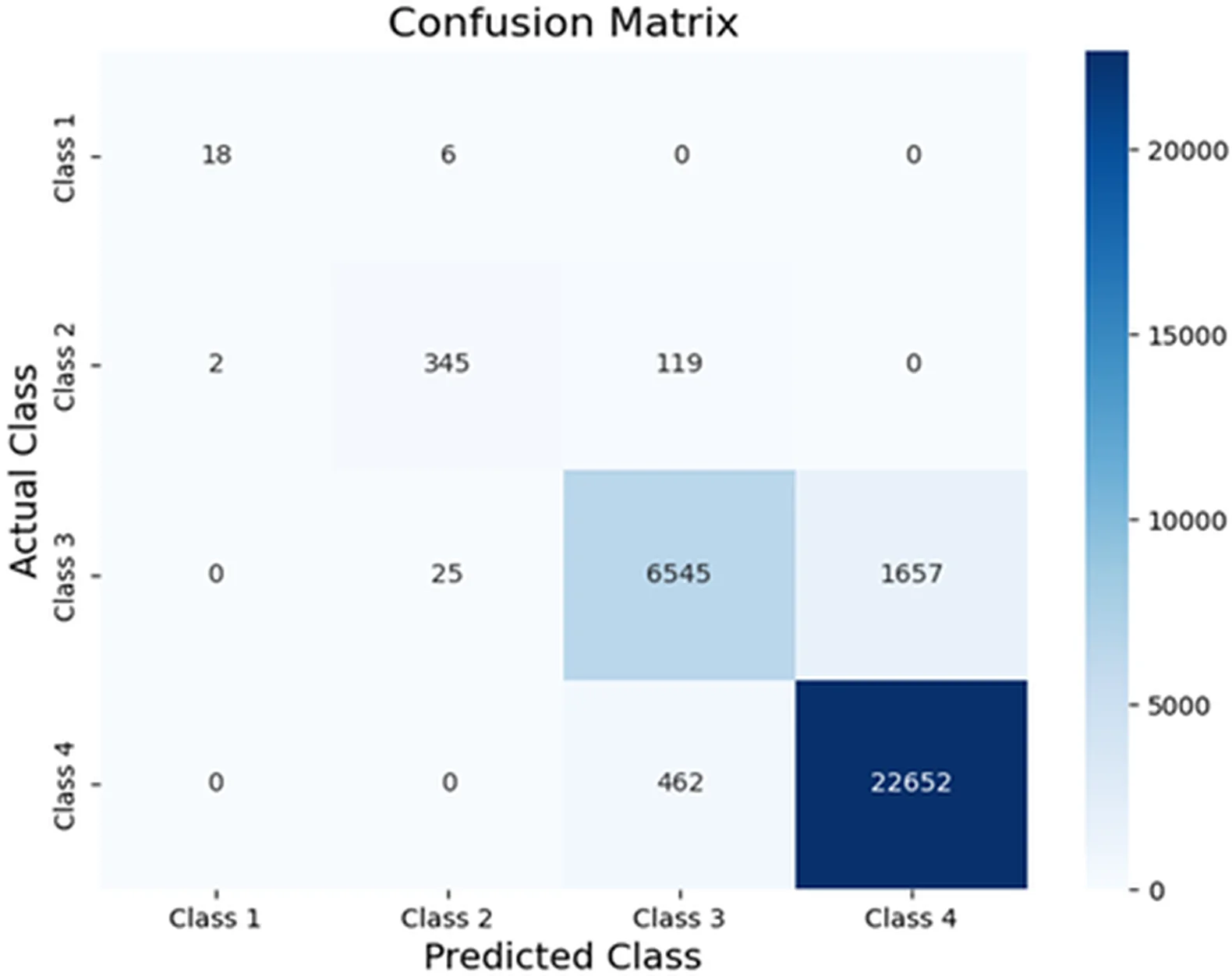
Confusion matrix.

## Discussion

The deep learning prediction framework integrating spatio-temporal features proposed in this study effectively solves the problems of focusing on static data and insufficient spatio-temporal feature capture in traditional traffic accident risk prediction, providing a new method path for expressway accident risk prediction. The CNN-BiLSTM model combines the advantages of CNN in spatial feature extraction and BiLSTM in time series processing, effectively capturing the spatio-temporal dynamic features of traffic data. Its high prediction accuracy is significantly better than single neural network models and traditional hybrid models, with moderate running time, achieving a good balance between prediction accuracy and computational efficiency.

The improvement in prediction accuracy can be attributed to the matching between the model structure and the spatio-temporal evolution of expressway accident risk. The CNN module extracts local traffic-interaction features from the vehicle trajectory data, while the bidirectional LSTM simultaneously captures the forward and backward temporal dependencies before and after a risk event. Because accident risk is influenced by both the evolution of traffic flow before the accident and the change of traffic state after the accident, the bidirectional design provides more complete temporal information than unidirectional models and therefore improves prediction accuracy.

The comparison with the CNN-LSTM model further supports this interpretation. CNN-LSTM uses a unidirectional LSTM and can only exploit historical information; during periods of severe sequence fluctuation, it cannot simultaneously refer to the subsequent traffic state, which leads to insufficient fusion of spatial features and temporal dependencies. This explains why the CNN-LSTM model shows obvious fitting deviation in the experimental results, whereas CNN-BiLSTM achieves the lowest errors with moderate running time.

The “PCA + FCM” two-level accident risk classification model constructed in this study effectively solves the data processing problem of multi-source heterogeneous traffic features, eliminates variable collinearity through PCA dimensionality reduction, realizes fuzzy division of accident risk using FCM algorithm, breaks through the limitation of fuzzy risk boundaries in traditional methods, and reveals the key causation mechanism of different risk levels through cluster center feature inversion, providing clear target variables for model construction and theoretical basis for traffic management departments to formulate targeted prevention and control measures.

This study still has certain limitations, pointing out the direction for subsequent research: first, the research data solely relies on the HighD natural driving dataset, and does not include multi-source heterogeneous data such as weather, road infrastructure and driver behavior characteristics, which is difficult to fully reflect the accident risk influencing factors in complex environments. Subsequent research can integrate multi-dimensional data, enrich model input features, and improve model applicability; second, model verification is only based on expressway scenarios, and the generalization ability in different traffic scenarios such as urban roads and ordinary national and provincial roads needs to be improved. Multi-scenario dataset training and model structure optimization are needed to enhance model adaptive ability; third, the study does not consider the impact of individual driver differences on accident risk, and only focuses on the single prediction task of accident risk. In the future, personalized risk modeling can be carried out combined with driving behavior data, and a multi-task learning framework can be explored to simultaneously realize joint prediction of accident risk, traffic flow and congestion, improving the comprehensive application value of the model.

In addition, the model training and verification of this study are based on historical trajectory data. In practical application, there is a certain delay in the collection and transmission of real-time traffic data. Subsequent research can combine edge computing, Internet of Things and other technologies to realize real-time collection of traffic data and online prediction of the model, improving the engineering application ability of the model; at the same time, advanced algorithms such as attention mechanism and graph neural network can be introduced to further optimize the CNN-BiLSTM model, enhance the model’s ability to capture key spatio-temporal features, and further improve prediction accuracy.

GNN-based models, spatio-temporal attention models and Transformer-based models have shown strong performance in regional traffic flow prediction by exploiting graph topology, adaptive feature weighting or global temporal modeling. However, their direct application to the present vehicle-level accident-risk task is not straightforward. The HighD trajectory dataset provides microscopic vehicle interactions without road-network topology or multi-source external information, and the short 0–4 s and 4–10 s windows emphasize localized temporal evolution rather than long-range network-level dependence. The proposed CNN-BiLSTM combines local feature extraction and bidirectional temporal modeling, and it achieved the best accuracy among the compared models under identical experimental conditions with moderate complexity. Future work will further evaluate GNN-based and attention-based models when road-network graph data and multi-source information become available.

It should be noted that the selection of the 4–10 s pre-accident feature window is primarily based on the TTC risk threshold (4 s) and empirical experience regarding car-following stability (10 s), rather than a systematic sensitivity analysis of alternative window lengths. Although the 0–4 s accident window is statistically justified by the frame distribution analysis, the optimal length of the pre-accident feature window warrants further investigation. Future work will explore adaptive window selection methods or systematic sensitivity analyses across different time horizons to enhance the robustness of feature extraction.

The relationship between the TTC-based event-screening criterion and the final risk labels was further considered from the perspective of model training. A direct TTC-interval labeling strategy would create a strong deterministic relationship between TTC and the target variable because TTC is also included among the traffic-interaction features used for prediction. Such a strategy could make the classifier rely disproportionately on TTC and could reduce its ability to learn the joint contribution of vehicle speed, acceleration, headway, speed difference, and other interaction characteristics.

The PCA-FCM labeling strategy also affects the upper bound of the achievable prediction performance. First, because the six retained principal components account for 83.449% of the total variance, approximately 16.6% of the original variability is discarded, which constitutes an information-theoretic limit for classification based on the reduced features. Second, FCM produces continuous membership values, while supervised learning requires discrete labels; converting the fuzzy partition into hard labels inevitably introduces label noise near cluster boundaries and therefore constrains the maximum attainable accuracy. Third, the model uses the 4−10 s pre-accident feature window to predict the risk state in the 0−4 s accident window, so the temporal mismatch between the feature and label windows further limits the information available for prediction. Accordingly, the approximately 90% overall accuracy of the CNN-BiLSTM model, with class-level precision values of 0.90–0.93 and F1 scores of 0.82–0.96, should be interpreted under these constraints rather than as an unbounded ceiling. A quantitative estimation of this upper bound is left for future work.

In contrast, the present study uses TTC < 4 s only to identify candidate high-risk events and derives the final labels from the joint distribution of multiple traffic-interaction variables through PCA-FCM. The resulting labels therefore represent broader interaction states rather than fixed TTC intervals. This design reduces the risk that a single TTC threshold dominates the target definition and provides a more multidimensional basis for subsequent supervised learning. However, because TTC remains one of the variables contributing to the clustering process, a degree of statistical dependence between TTC and the derived labels is inherent in the current labeling framework and should be considered when interpreting the prediction results.

## Conclusions

This study systematically conducts research on expressway traffic accident risk prediction considering spatio-temporal effects from data preprocessing, spatio-temporal feature analysis, accident risk classification, model construction and optimization to model evaluation and application, and obtains the following core conclusions:

(1) Expressway traffic accident risk has significant spatio-temporal heterogeneity. Indicators such as rear vehicle speed/acceleration, headway, time to collision and number of lane changes are highly correlated with accident risk, and there are obvious collinearity problems between features. PCA dimensionality reduction can effectively retain core information, and FCM clustering can accurately divide accident risk into three levels: serious, general and minor, providing clear target variables for the prediction model.
(2) The proposed CNN-BiLSTM hybrid deep learning model integrates the spatial local feature extraction capability of CNN and the forward and backward long-term dependency capture capability of BiLSTM in time series, which can effectively handle the nonlinear spatio-temporal relationship in traffic data. After structure and hyperparameter optimization, the model prediction accuracy reaches 90%, which is significantly better than comparative models such as LSTM, BiLSTM and CNN-LSTM in quantitative indicators such as MSE, RMSE, MAE and MAPE, with moderate running time, balancing prediction accuracy and computational efficiency.
(3) The CNN-BiLSTM model performs excellently in accident risk classification prediction, with the prediction accuracy of serious, general, minor accidents and no accident state all reaching about 0.90, and the recall rate and F1 score are at a high level, which can accurately identify different levels of accident risks, providing a scientific basis for active safety prevention and control and rescue scheduling of traffic management departments.
(4) The “extraction-cleaning-integration” data preprocessing process designed for the HighD dataset effectively solves problems such as data abnormality and cross-file conflicts, providing a standardized processing method for traffic accident risk research; the hyperparameter optimization strategy based on grid search and time series cross-validation fully adapts to the characteristics of time series data, ensures the optimal configuration of model parameters, and provides a reference for the construction and optimization of similar deep learning models.

The research results provide an efficient accident risk prediction tool for intelligent traffic management, and have important practical guiding significance for optimizing driving behavior and improving expressway road safety. With the advancement of data collection technology and the continuous development of deep learning methods, the traffic accident risk prediction model integrating spatio-temporal effects will play a more important role in intelligent transportation systems, providing more comprehensive and efficient guarantee for road traffic safety.
